# scEVE: a single-cell RNA-seq ensemble clustering algorithm capitalizing on the differences of predictions between multiple clustering methods

**DOI:** 10.1093/nargab/lqaf073

**Published:** 2025-06-09

**Authors:** Yanis Asloudj, Fleur Mougin, Patricia Thébault

**Affiliations:** Univ. Bordeaux, CNRS, Bordeaux INP, LaBRI, UMR 5800, F-33400 Talence, France; Univ. Bordeaux, INSERM , BPH, U1219, F-33000 Bordeaux, France; Univ. Bordeaux, CNRS, Bordeaux INP, LaBRI, UMR 5800, F-33400 Talence, France; Univ. Bordeaux, INSERM , BPH, U1219, F-33000 Bordeaux, France; Univ. Bordeaux, CNRS, Bordeaux INP, LaBRI, UMR 5800, F-33400 Talence, France

## Abstract

Single-cell RNA sequencing measures individual cell transcriptomes in a sample. In the past decade, this technology has motivated the development of hundreds of clustering methods. These methods attempt to group cells into populations by leveraging the similarity of their transcriptomes. Because each method relies on specific hypotheses, their predictions can vary drastically. To address this issue, ensemble algorithms detect cell populations by integrating multiple clustering methods, and minimizing the differences of their predictions. While this approach is sensible, it has yet to address some conceptual challenges in single-cell data science; namely, ensemble algorithms have yet to generate clustering results with uncertainty values and multiple resolutions. In this work, we present an original approach to ensemble clustering that addresses these challenges, by describing the differences between clustering results, rather than minimizing them. We present the scEVE algorithm, and we evaluate it on 15 experimental datasets, and up to 1200 synthetic datasets. Our results reveal that scEVE outperforms the state of the art, and addresses both conceptual challenges. We also highlight how biological downstream analyses will benefit from addressing these challenges. We expect that this work will provide an alternative direction for developing single-cell ensemble clustering algorithms.

## Introduction

Single-cell RNA sequencing, or scRNA-seq, is a technique used to measure the transcriptomes (i.e. the global gene expression) of individual cells within a biological sample. This technique was introduced by Tang *et al.* in 2009 [1] to capture the transcriptome of a single mouse blastomere. Ever since, a diversity of automated sequencers with enhanced protocols have emerged, and they are routinely employed by the community to study biological tissues at the cell resolution. For example, in 2016, Baron *et al.* [2] used scRNA-seq to describe the cell composition of human and mouse pancreases. The next year, Darmanis *et al.* [3] used it to study human glioblastoma tumors. Reviews ranging from 2017 to 2023 [4–6] propose a broader overview of the scRNA-seq technologies and their applications. Remarkably, they also establish scRNA-seq technologies as a pivotal tool for modern biology.

Briefly, scRNA-seq technologies generate a matrix reporting the transcriptomes of every cell sampled within a tissue. This matrix is used to study the cellular diversity of the tissue, and is referred to as a scRNA-seq dataset. By performing computational analyses on this dataset, multiple biological questions can be answered. For instance, to study cell differentiations, a trajectory inference analysis can be conducted. Alternatively, to study cell abundances, best practices recommend carrying out a compositional analysis [5, 6]. Although these analyses answer distinct biological questions, they all rely on the same prior clustering analysis [4–7].

A scRNA-seq clustering analysis identifies homogeneous populations (i.e. “clusters”) of cells by leveraging the similarity of their transcriptomes. Put simply, it groups similar cells together. Due to its fundamental role in scRNA-seq analyses [7], a myriad of clustering methods has been developed; in January 2024, scRNA-tools [8] surveyed more than 375 clustering methods applicable to scRNA-seq datasets. Remarkably, 7 years prior, only 10 methods were reported. Obviously, this booming diversity of methods makes it difficult to navigate the scRNA-seq landscape, for newcomers and experts alike. Fortunately, to identify the best available clustering methods, their performances are regularly compared in benchmark studies (e.g. [9–11]). Unfortunately, these benchmarks show that the clustering performance of each method is impacted by user-specific choices [10], and by characteristics of the data that are rarely known *a priori*, such as the size or the type of the cell populations [9, 11].

In other words, the results of a clustering analysis are dependent on the clustering method used; the performance of this clustering method is affected by specific data settings; and these settings are unknown prior to conducting the clustering analysis. Consequently, the results of a clustering analysis are always biased by the method used.

To address this bias, ensemble clustering algorithms (“ensemble algorithms”) have been developed (e.g. [12–14]). Succinctly, an ensemble algorithm generates a set of different clustering results, and it integrates them together to output a unique consensus clustering result [15]. Thus, by leveraging multiple methods (each being sensitive to different data settings), ensemble algorithms can effectively address the methodological bias of clustering analyses. Incidentally, they also exploit the dataset more extensively than a single clustering method would.

In their 2011 review on ensemble algorithms, Vega-Pons and Ruiz-Schulcloper [15] defined a taxonomy of approaches to integrate multiple clustering results together. They termed these approaches “consensus functions,” and they classified them into two groups: (i) functions based on median partition and (ii) functions based on object co-occurrences. On one hand, median partition-based functions output the most average clustering result with regards to a set of input clustering results. This is usually done by optimizing an objective function that quantifies the similarity between the consensus and the input clustering results (e.g. [14, 16, 17]). On the other hand, a function based on object co-occurrences quantifies the number of times each pair of cells is grouped together (in a set of input clustering results), and it performs a final clustering analysis on these similarity-like measurements (e.g. [12, 13, 18]). Intuitively, in both cases, the consensus function attempts to minimize the differences between input clustering results.

In this work, we explore an alternative approach to integrate multiple clustering results together. We hypothesize that the differences in a set of input clustering results are informative, and we propose to describe them and leverage them (instead of minimizing them) in order to identify clusters robust to the method used, and to prevent over-clustering (i.e. the identification of false cell populations). To verify this hypothesis, we have developed scEVE, an ensemble algorithm that embraces this novel philosophy. Instead of functions based on median partition or object co-occurrences [15], scEVE uses only fundamentals from graph and ensemble theories to identify robust clusters, and to quantify their robustness. Incidentally, it effectively tackles two grand challenges yet unaddressed in single-cell data science [19], namely (i) the need to study cells at multiple resolutions and (ii) the need to quantify the uncertainty of the results.

In this work, the scEVE algorithm is presented and evaluated. First, we use scEVE to carry out an in-depth clustering analysis of a human glioblastoma scRNA-seq dataset [3]. This application showcases the conceptual benefits of our algorithm. Then, we compare the performances of scEVE with the ones of the clustering methods it integrates, as well as the ones of state-of-the-art scRNA-seq ensemble algorithms. This comparison is conducted on 15 experimental datasets, and up to 1200 synthetic datasets.

## Materials and methods

**Figure 1.**
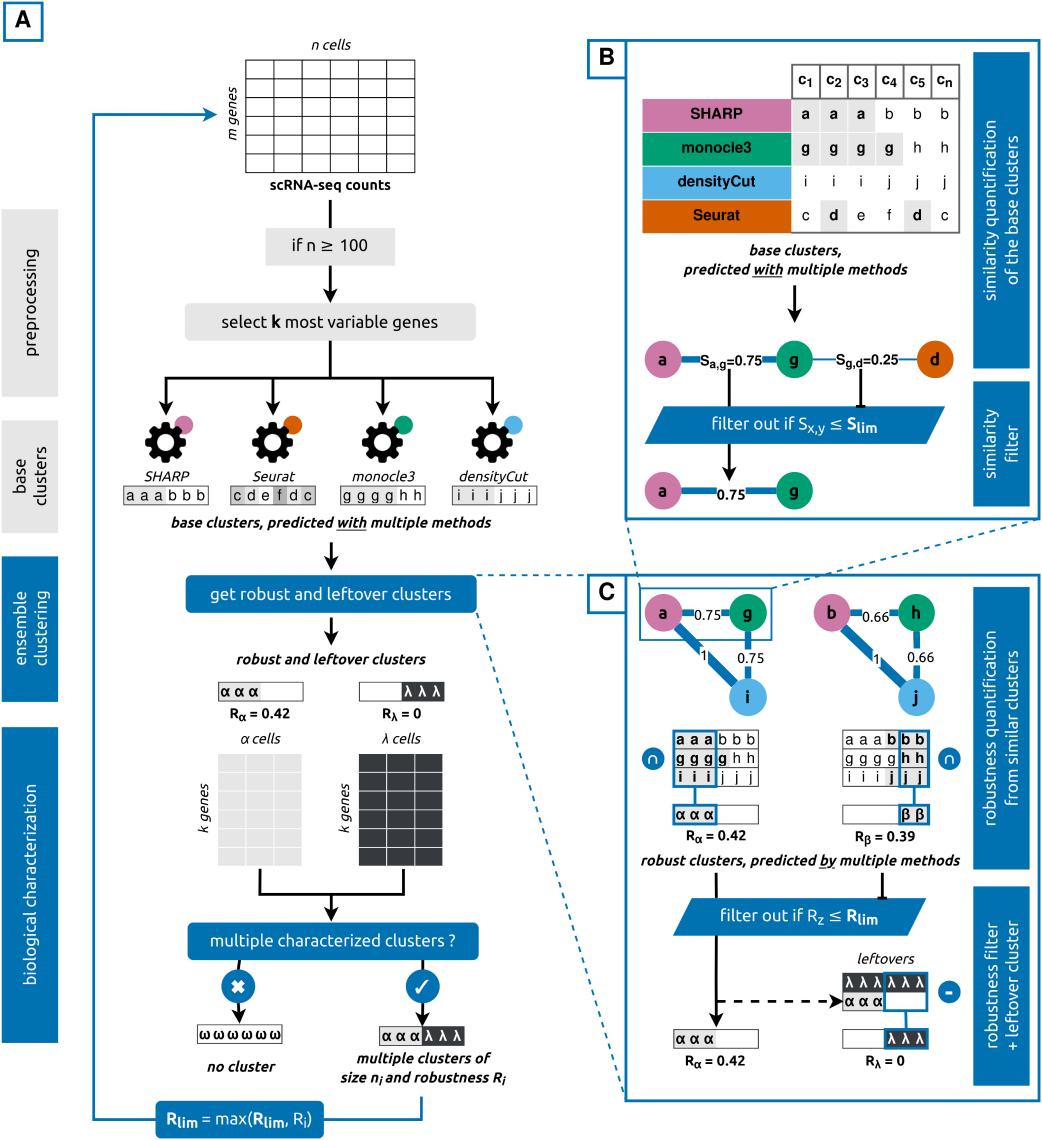
Overview of the scEVE algorithm. (**A**) Base clusters with multiple clustering methods, and biological characterization of robust clusters. A single-cell count matrix is preprocessed and fed to multiple clustering methods to generate base clusters. From these, robust and leftover clusters are predicted (see panel C) and characterized. As a result, biologically distinct clusters are identified and returned. They will eventually be recursively subdivided, provided this increases the robustness of the clustering analysis. (**B**) Pairwise similarity between base clusters. Cells at the intersection of two base clusters *x* and *y* are used to quantify the similarity *S*_*x*,*y*_. If this similarity is strong (*S*_*x*,*y*_ > *S*_lim_), it is leveraged downstream to identify robust clusters. (**C**) Robust clusters from pairwise similarities. Cells grouped together by multiple clustering methods form a robust cluster *z*. Its robustness *R*_*z*_ is quantified by exploiting the pairwise similarities computed previously (see panel B). If, and only if, this robustness is high (*R*_*z*_ > *R*_lim_), the cluster will be characterized downstream. The remaining cells form a leftover cluster *λ*, with *R*_*λ*_ = 0, which will also be characterized downstream.

scEVE is a recursive ensemble clustering algorithm, fully implemented in R [20]. At each recursion, scEVE applies multiple clustering methods on a pool of cells to generate “base clusters.” From these base clusters, an original pairwise similarity metric is computed. Then, by leveraging these measurements, the scEVE algorithm identifies “robust clusters” (i.e. clusters of cells that are grouped together by multiple clustering methods), and it quantifies their robustness. Eventually, it applies a filter based on marker genes to ensure that the robust clusters are distinct and informative for the downstream biological analyses. The resulting clusters are automatically subdivided, on the condition that the robustness of the clustering analysis increases by doing so. We summarize the scEVE algorithm in Fig. 1, and we describe it more extensively in the “scEVE algorithm” section. In the “Evaluation” section, we present the approach we employed to evaluate scEVE.

### scEVE algorithm

#### Selection of multiple clustering methods

scEVE leverages multiple clustering methods, several times. Consequently, the clustering methods it integrates should be methodologically different and computationally efficient. To identify such methods, we have used the benchmark proposed by Yu *et al.* in 2022 [11]. In this work, the authors have benchmarked and classified 14 different scRNA-seq clustering methods into four different groups: (i) methods based on community detection, (ii) methods based on inter/intra-cluster similarities, (iii) methods based on stability metrics, and (iv) methods based on eigenvector metrics. Out of the 14 methods benchmarked, 4 were very efficient computationally [11]: monocle3 [21], densityCut [22], Seurat [23], and SHARP [24]. These four methods cover three different clustering approaches, and their clustering performance is ranked from best to worst (Table 1). Accordingly, they were selected to generate the base clusters of scEVE.

**Table 1.** Overview of the four clustering methods integrated in scEVE

Method	Clustering approach	Rank/14	Reference
monocle3	Community detection	1	[21]
Seurat	Community detection	5	[23]
densityCut	Stability metrics	13	[22]
SHARP	Inter/intra-cluster similarities	14	[24]

*Source*: Yu *et al.* [11].

#### Base clusters with multiple clustering methods

scEVE takes a single-cell count matrix as input. Following best practices in scRNA-seq analyses [6], *k* highly variable genes are selected, and their expression is used to generate base clusters with multiple clustering methods (Fig. 1A). These highly variable genes are selected with the function FindVariableFeatures() of the Seurat library [23]. To follow best practices [6] and remain computationally efficient, we set *k* to 1000.

The clustering methods are run with their default parameters, to reflect their average usage. By default, the SHARP [24] clustering method expects a hundred cells (sncells = 100). Consequently, to ensure that the method runs correctly, scEVE will not attempt to cluster a pool of cells with *n* < 100 cells.

To further guarantee the correct execution of the clustering methods, the count data are transformed into log_2_(TPM) (transcript per million) prior to the densityCut [22] clustering analysis. This transformation simulates the inputs expected by densityCut, and is performed using the calculateTPM() function of the scater library [25].

#### Pairwise similarity between base clusters

scEVE identifies strong pairwise similarities in the base clusters (Fig. 1B). They are eventually exploited to identify robust clusters, predicted by multiple methods (Fig. 1C).

**Figure 2.**
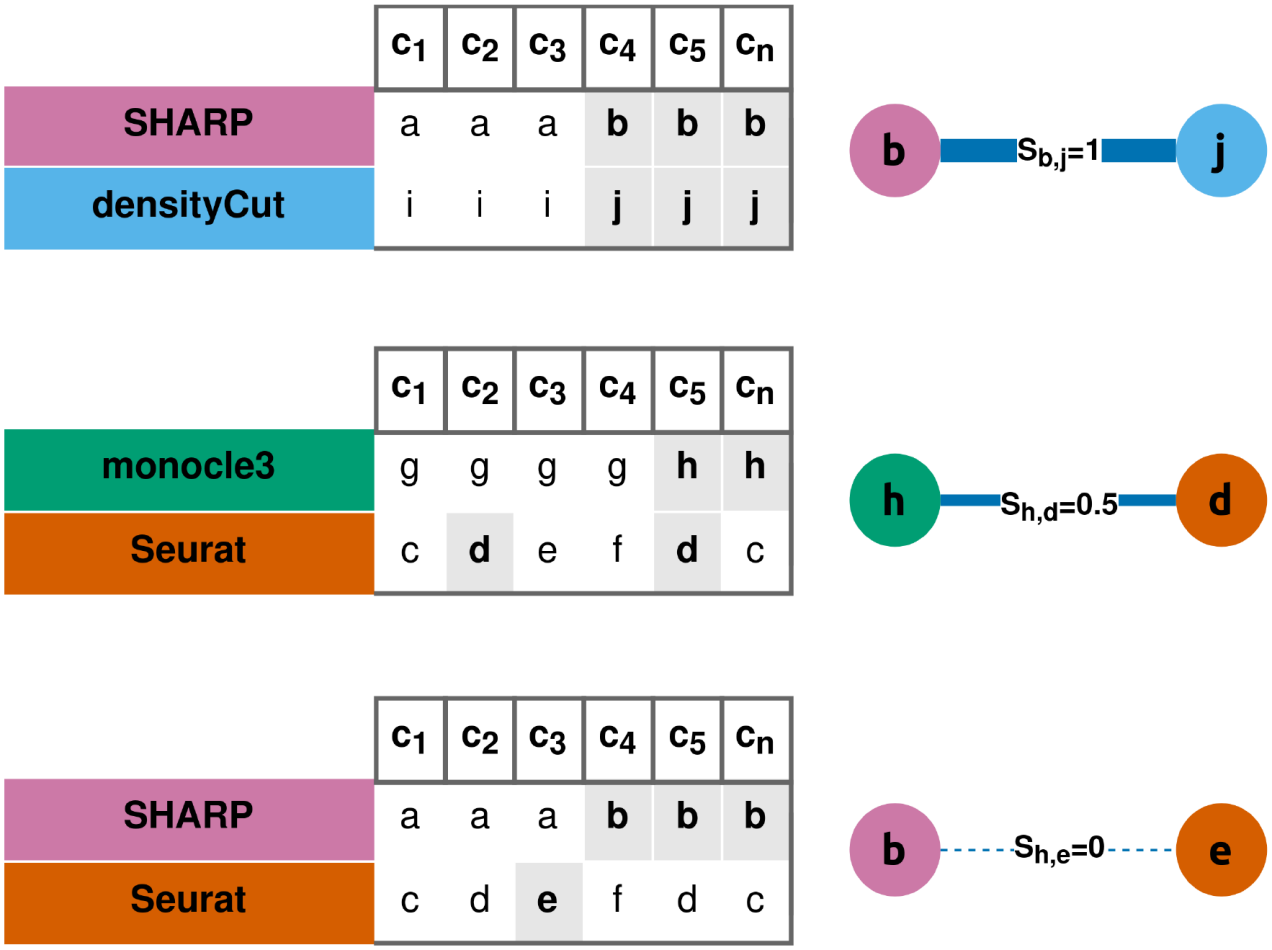
Pairwise similarity of two base clusters (toy example). Letters represent base clusters, predicted with multiple clustering methods. For instance, the base cluster *d* is composed of the cells *c*_2_ and *c*_5_, and it is predicted by Seurat. The cells at the intersection of two base clusters *x* and *y* are used to quantify the similarity *S*_*x*,*y*_. It ranges from 0 to 1. If *x* and *y* are identical, *S*_*x*,*y*_ = 1. If *x* and *y* are disjoint, *S*_*x*,*y*_ = 0.

The pairwise similarity between two base clusters *x* and *y* is noted *S*_*x*,*y*_. It corresponds to the minimal proportion of cells shared by the two clusters (Fig. 2), and it is calculated as follows (Equation 1):


(1) \begin{eqnarray*}
S_{x,y} = {\rm min}\left(\frac{N_{x \cap y}}{N_{x}}, \frac{N_{x \cap y}}{N_{y}}\right){\rm ,}
\end{eqnarray*}

where *S_x_*_,*y*_ is the similarity between two base clusters *x* and *y*, *N_x_*_∩*y*_ is the number of cells in both clusters *x* and *y*, and *N_x_* is the number of cells in the cluster *x*.

To be considered strong, pairwise similarities must exceed a threshold *S*_lim_. We set *S*_lim_ to 0.5, to ensure that *S*_*x*,*y*_ is considered a strong pairwise similarity if, and only if, two clusters *x* and *y* share the majority of their cells.

In practice, to measure the similarity *S*_*x*,*y*_, a frequent itemset mining algorithm is employed. Specifically, we use the Apriori algorithm [26], implemented in the function apriori() of the arules library [27]. Briefly, frequent itemset mining algorithms quantify the co-occurrences of items across multiple sets. They are computationally efficient, and they are able to measure a variety of metrics. One of these metrics is the confidence conf(*A* → *B*), which corresponds to the proportion of sets containing item *A* that also contain item *B*. By considering cells as sets and their respective clusters as items, we observe that ${\rm conf}(x \rightarrow y)={N_{x \cap y}}/{N_{x}}$. Hence, *S*_*x*,*y*_ can effectively be measured with a frequent itemset mining algorithm. Readers interested in more information regarding the frequent itemset framework and its applications to bioinformatics can refer to Naulaerts *et al.* [28].

#### Robust clusters from pairwise similarities

scEVE employs graph theory to model base clusters as vertices, and pairwise similarities as weighted edges. Edges corresponding to weak pairwise similarities are filtered out, and as a result, multiple subgraphs—i.e. multiple connected components—are generated. scEVE exploits each subgraph (i) to identify a robust cluster of cells and (ii) to quantify its robustness. It also aggregates every cell unassigned to a subgraph into an additional cluster, called the “leftover cluster” (Fig. 1C).

**Figure 3.**
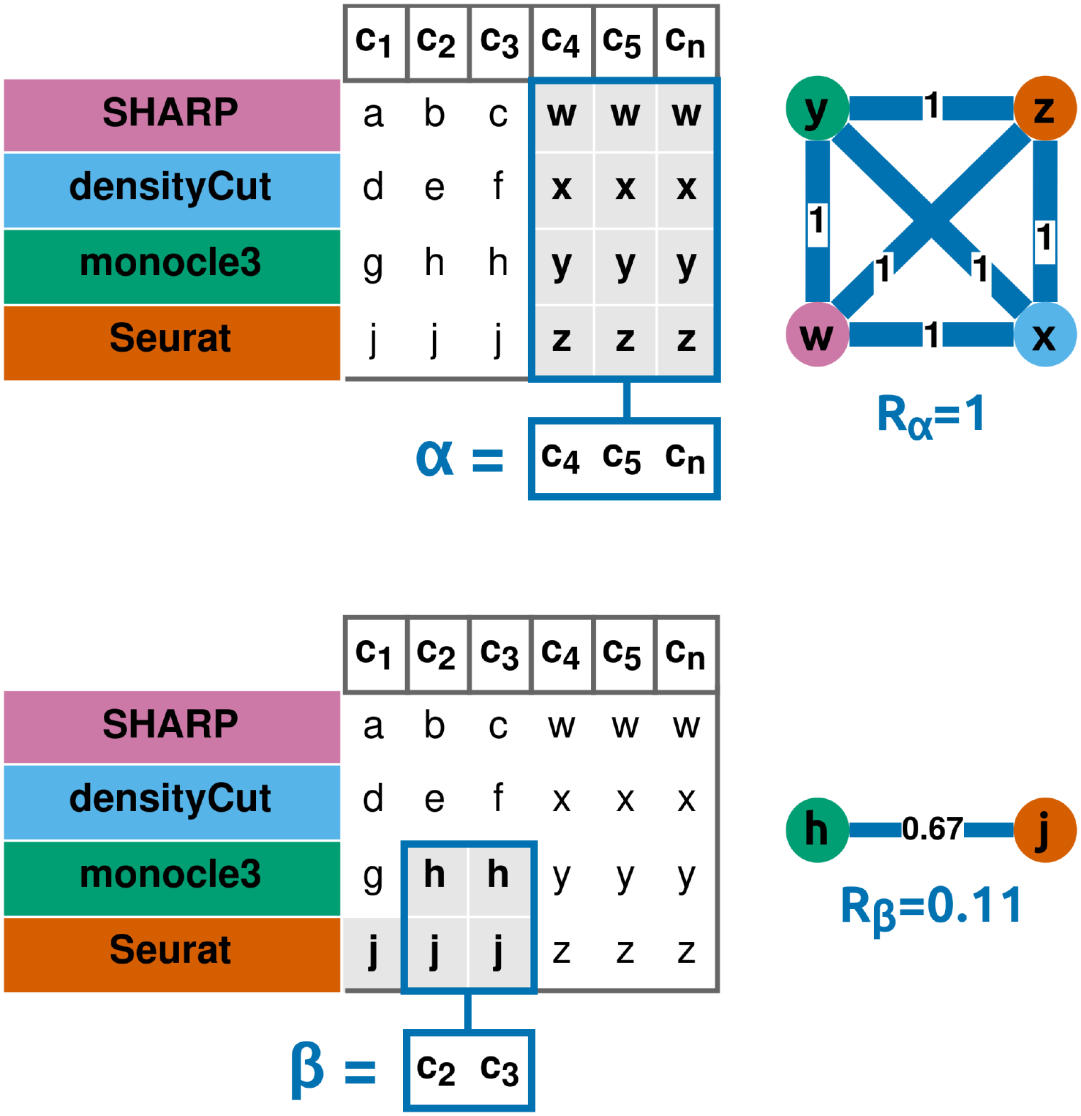
Robustness of a cluster (toy example). Latin letters (e.g. *a*, *b*) represent base clusters, predicted with multiple clustering methods. For instance, the base cluster *h* is composed of the cells *c*_2_ and *c*_3_, and it is predicted by monocle3. Greek letters (*α*, *β*) represent robust clusters, i.e. cells grouped together by multiple clustering methods. For instance, *β* is composed of the cells *c*_2_ and *c*_3_, grouped together by monocle3 and Seurat, and *R*_*β*_ is its robustness. The robustness ranges from 0 to 1, and it is equal to 1 if, and only if, every clustering method predicts exactly the same cluster.

If a subgraph exists, it indicates that some base clusters are very similar, i.e. multiple methods have grouped the same cells together. Consequently, we define a robust cluster as a set of cells grouped together in all the base clusters of a subgraph. To quantify the robustness of this cluster, the structure of the subgraph is also leveraged: still according to graph theory, if all *M* clustering methods predicted exactly the same cluster, the sum of the edges of the resulting subgraph would yield ${M(M-1)}/{2}$. By comparing this theoretical sum with the one obtained experimentally—i.e. by calculating the weighted density of the subgraph—we can measure the robustness of a new cluster *z* (Fig. 3). This robustness is named *R*_*z*_, and is calculated as follows (Equation 2):


(2) \begin{eqnarray*}
R_{z} = \frac{\sum _{x,y}{S_{x,y}}}{\frac{M(M-1)}{2}}{\rm ,}
\end{eqnarray*}

where *S_x,y_* is the similarity between two base clusters *x* and *y*, *R_z_* is the robustness of a robust cluster *z*, and *M* is the total number of clustering methods used.

To guarantee the validity of the robust clusters, a robustness filter is applied, based on a threshold *R*_lim_. It corresponds to the minimum expected robustness of a cluster, if the majority of methods were to predict it. It is calculated automatically by the algorithm according to Equation 3, and is thus equal to 0.25 with four clustering methods integrated.


(3) \begin{eqnarray*}
R_{{\rm lim}} = \frac{S_{{\rm lim}} * \frac{m(m-1)}{2}}{\frac{M(M-1)}{2}}{\rm ,}
\end{eqnarray*}

where *R*_lim_ is the robustness threshold, *S*_lim_ is the similarity threshold, *m* is the number of clustering methods required to have a majority, and *M*is the total number of clustering methods used.

Finally, a “leftover cluster” is created by grouping together all the cells unassigned to a robust cluster. Because the existence of this leftover cluster is caused by a lack of agreements between the clustering methods, its robustness is set to 0.

#### Biological characterization of robust clusters

To ensure that the robust clusters and the leftover cluster are biologically distinct and well characterized for the downstream analyses, scEVE attempts to detect marker genes within these clusters. Based on a meta-analysis conducted by Fischer and Gillis in 2021 [29], we define marker genes as genes expressed 16 times more in a cluster than in the rest of the pool (i.e. log_2_ fold change >4). These genes are detected by using the function FindMarkers() of the Seurat library [23].

To define poorly and well-characterized clusters, we again rely on the work of Fischer and Gillis [29] to consider that a cluster is well-characterized if 10 marker genes are detected in it. After an initial attempt to detect marker genes, poorly characterized clusters are merged within the leftover cluster, and a new characterization attempt is conducted. Following this analysis, if new characterized clusters are identified, the initial pool of cells is clustered. Otherwise, scEVE does not predict any cluster in order to avoid over-clustering (Fig. 1A).

#### Recursive and transparent prediction of clusters.

After a clustering recursion, scEVE automatically attempts to subdivide the cell clusters predicted. This subdivision is conducted by running a new clustering recursion on each cell cluster, starting from the generation of “base clusters with multiple clustering methods,” and ending with the “biological characterization of robust clusters.”

To further prevent over-clustering, the robustness threshold *R*_lim_ increases dynamically with each recursion as follows: for any predicted cluster *z*, a subcluster *i* is predicted if *R*_*i*_ > max(*R*_lim_, *R*_*z*_). By raising the robustness threshold, we ensure that subclusters are predicted if, and only if, the clustering methods integrated into scEVE agree on their existence more than that of their parent cluster.

Finally, because numerous aspects of the data are evaluated during a clustering recursion, scEVE automatically generates plots to help monitor the clustering analysis. These plots further improve the transparency of our algorithm, and they are generated at each recursion by using the SCpubr [30], ggVennDiagram [31, 32], and ggplot2 [33] libraries.

### Evaluation

#### Selection of experimental scRNA-seq datasets

In order to evaluate the performance of the scEVE algorithm, we have applied it on a variety of publicly available scRNA-seq datasets. These datasets were extracted from two different scRNA-seq databases. The first one, TMExplorer [34], includes datasets sequenced from multiple mammalian tumors. The second one, the Hemberg group collection [35], includes datasets commonly used in the scRNA-seq literature, and sequenced from a variety of mammalian tissues.

To select the datasets used in our experiments, we had three inclusion criteria. Naturally, (i) the dataset had to be associated with a ground truth and readily available. Furthermore, to respect the specific features of the clustering methods integrated into scEVE (see the “Base clusters with multiple clustering methods” section), the dataset had to be (ii) a count matrix, (iii) with *n* > 100 cells.

According to these three criteria, we were able to extract 15 datasets. They are sequenced from nine different human and mouse tissues, with five distinct sequencing protocols, and the magnitude of their sizes ranges from hundreds of cells to tens of thousands of cells. We argue that the properties of our datasets are sufficiently diverse to conduct an extensive benchmark, and we summarize them in Table 2.

**Table 2.** Overview of the 15 experimental scRNA-seq datasets used

Dataset	Cells	Clusters	Genes	Protocol	Accession	Reference
Peng_HumPDAC	57 530	10	24 005	10x Genomics	CRA001160	[36]
Lambrechts_HumNSCLC	51 775	17	22 180	10x Genomics	E-MTAB-6149, E-MTAB-6653	[37]
VanGalen_HumAML	22 600	17	27 899	Seq-Well	GSE116256	[38]
Gillen_HumEPN	18 456	18	23 580	10x Genomics	GSE125969	[39]
JerbyArnon_HumMLM	6879	9	23 686	SMART-Seq2	GSE115978	[40]
Baron_HumPan_3	3605	14	20 125	inDrop	GSE84133	[2]
Darmanis_HumGBM	3589	7	23 460	SMART-Seq2	GSE84465	[3]
Baron_HumPan_1	1937	14	20 125	inDrop	GSE84133	[2]
Baron_HumPan_2	1724	14	20 125	inDrop	GSE84133	[2]
Tasic_MouBra	1679	18	24 057	SMARTer	GSE71585	[41]
Baron_HumPan_4	1303	14	20 125	inDrop	GSE84133	[2]
Baron_MouPan_2	1064	13	14 878	inDrop	GSE84133	[2]
Baron_MouPan_1	822	13	14 878	inDrop	GSE84133	[2]
Li_HumCRC_a	561	9	55 186	SMARTer	GSE81861	[42]
Li_HumCRC_b	364	7	57 241	SMARTer	GSE81861	[42]

*Abbreviations*: Hum: human; Mou: mouse; Pan: pancreas; Bra: brain; PDAC: pancreatic ductal adenocarcinoma; NSCLC: non-small-cell lung cancer; AML: acute myeloid leukemia; EPN: ependymoma; MLM: melanoma; GBM: glioblastoma; CRC: colorectal cancer.

*Sources*: Christensen *et al.* [34] and Kiselev *et al.* [35].

#### Generation of synthetic scRNA-seq datasets

Additionally to the experimental datasets, we have also used synthetic datasets to evaluate scEVE under multiple controlled settings.

The datasets were generated using the SPARSim [43] simulation method, whose performance, scalability, and applicability ranked highest in the benchmark proposed by Cao *et al.* in 2021 [44]. Briefly, to generate a scRNA-seq dataset with multiple cell clusters, SPARSim takes an original dataset as input, and modifies it by introducing biological variability and differentially expressed genes. Accordingly, our synthetic datasets were generated with an input dataset of peripheral blood mononuclear cells (PBMC 10x Genomics), which is readily available with SPARSim, and commonly used in the scRNA-seq literature.

To evaluate our algorithm under different settings, we have generated synthetic datasets with *N* = 10 000 cells, and we have altered the number of clusters, their sizes, and their transcriptomes. The resulting datasets are composed of 1–10 clusters, with cluster sizes balanced or not, and cluster transcriptomes related or not. For each combination of settings, up to 30 replicates were generated, and a total of 1200 datasets were generated.

By generating datasets with imbalanced cluster sizes, we emulated the existence of rare cell populations. While each cluster has the same size in balanced datasets, their size follows a geometric distribution in imbalanced datasets (Equation 4). Accordingly, the sizes of five clusters would be {2000, 2000, 2000, 2000, 2000} in a balanced dataset, and {5161, 2580, 1290, 645, 322} in an imbalanced dataset.


(4) \begin{eqnarray*}
n_{i} = \frac{N}{2^{i}} + \frac{N}{2^{k}} * \frac{2^{k-i}}{2^{k-1}}{\rm ,}
\end{eqnarray*}

where *n*_*i*_ is the number of cells in the synthetic cluster *i*, *i* is the rank of a synthetic cluster, ordered by descending size, *k* is the number of clusters in the synthetic dataset, and *N* is the number of cells in the synthetic dataset.

**Figure 4.**
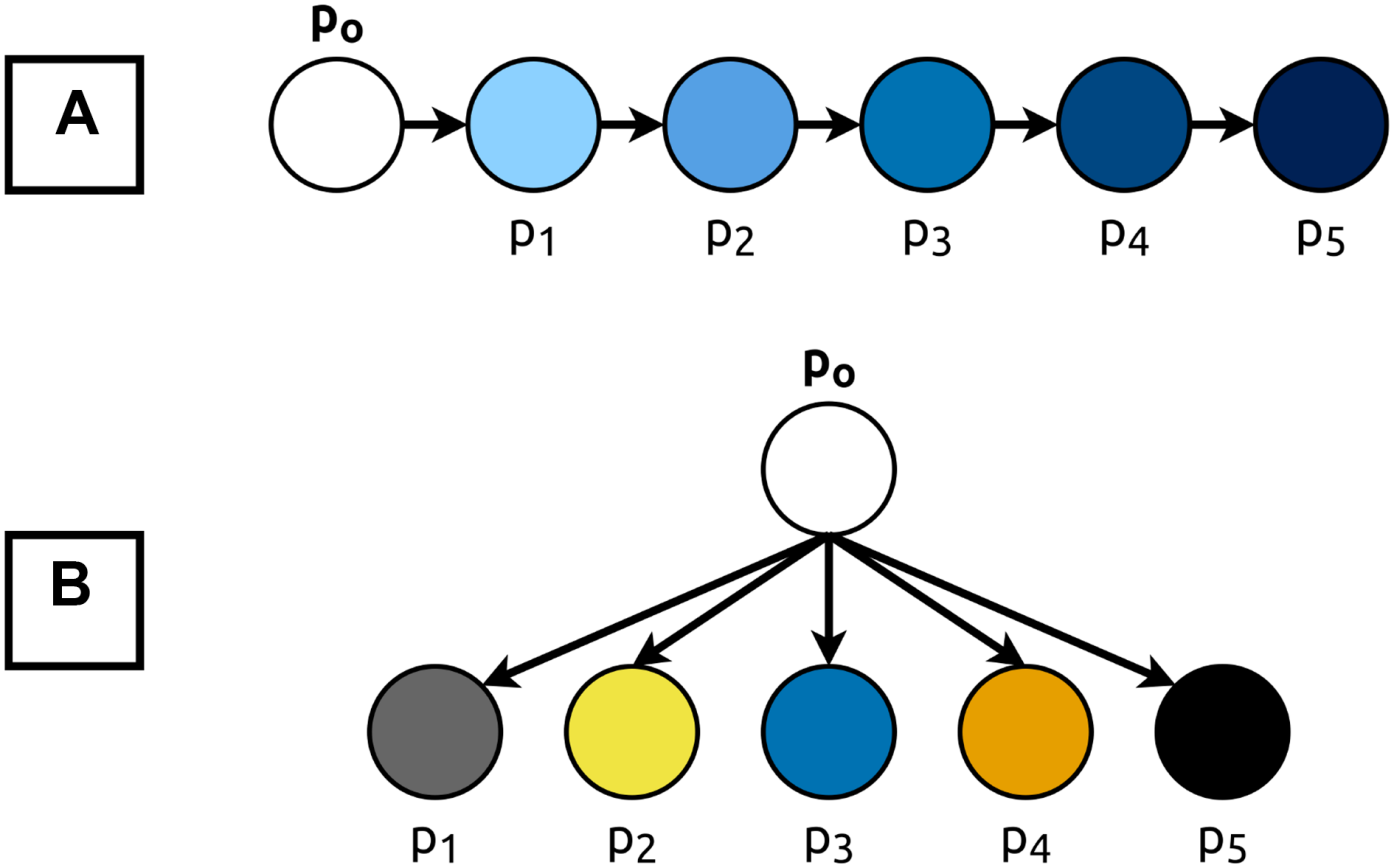
Generation of (**A**) related and (**B**) unrelated synthetic datasets. Synthetic cell populations *p*_1_ to *p*_5_ are generated from an input population *p*_*o*_. The circles represent cell populations, and the arrows represent SPARSim [43] modifications.

We also emulated the existence of related cell populations (e.g. CD4^+^ and CD8^+^ T cells) by generating related datasets. In these datasets, the parameters of the input dataset are modified only once to generate a cluster *n*, and the cluster *n* + 1 is generated by modifying the parameters of the cluster *n*. Consequently, the clusters’ transcriptomes are all related to each other, to varying degrees. Oppositely, in unrelated datasets, clusters are all generated by modifying the parameters of the input dataset, with a different seed each time (Fig. 4).

#### Calculation of performance metrics

The clustering performance of scEVE was measured with four different metrics: the normalized mutual information (NMI) [17], the Silhouette index (SI) [45], the adjusted Rand index (ARI) [46], and the neighborhood purity (nPurity). These four metrics were calculated using the aricode [47] and bluster [48] libraries.

The ARI [46] and the NMI [17] are two extrinsic clustering metrics: they measure the similarity between two sets of clusters. Their values are proportional to this similarity, and it is equal to 1 if the two sets are identical. Their equations are reported in the Supplementary data. We use the ground truth clusters of every dataset to measure these metrics.

The SI [45] and the nPurity are two intrinsic clustering metrics that exploit cell gene expression to assess cluster cohesion. In short, they quantify the similarity of cell transcriptomes within a cluster, with regards to the dissimilarity of cell transcriptomes between clusters. Their values are proportional to these two properties, with a maximum of 1, and can be averaged across all cells to evaluate cluster predictions of a dataset. The SI equation is reported in the Supplementary data, and we refer readers interested in the nPurity metric to the function neighborPurity() of the bluster [48] library.

The four aforementioned metrics are used to evaluate the “leaf clusters” predicted by scEVE. We define a leaf cluster, as a cluster that scEVE was not able to subdivide (i.e. the clusters predicted at the maximum resolution). We evaluate the performance of scEVE with (labeled scEVE) and without leftover clusters (labeled scEVE*).

Finally, scEVE’s computational performance (i.e. maximum memory usage and algorithm computation time) was also measured in our experiments.

#### Comparison with the state of the art

To evaluate scEVE and benchmark it against state-of-the-art scRNA-seq clustering methods, we have compared its performance with four clustering methods and three ensemble algorithms.

First, we have compared scEVE with the four clustering methods it integrates: densityCut [22], monocle3 [21], Seurat [23], and SHARP [24]. The clustering analyses were conducted as described in the “Base clusters with multiple clustering methods” section, except that we selected 5000 variable genes (instead of 1000) for preprocessing (which corresponds to best practice [6] when computation costs are not a limitation).

We have also compared scEVE to other scRNA-seq ensemble algorithms. These algorithms were first selected according to three criteria: (i) they leveraged base clusters generated with multiple methods, and were (ii) flexible and (iii) unsupervised. Consequently, we excluded algorithms leveraging base clusters generated from different data representations [15], such as random projections of the data [49]. We also excluded algorithms developed to integrate a specific combination of clustering methods [50], or algorithms integrating supervised clustering methods [51].

In practice, the scRNA-seq ensemble algorithms were selected by manually exploring the literature. Specifically, we discovered the SAFE algorithm [14] by searching for “single-cell ensemble clustering” on Google Scholar; and because SAFE was the first scRNA-seq algorithm to integrate multiple clustering methods (according to its authors), we searched for all algorithms that cited it. For each algorithm meeting our inclusion criteria, similar searches were carried out. Finally, seven ensemble algorithms were selected: EC-PGMGR [52], GRACE [53], RSEC [13], SAFE [14], SAME [16], scEFSC [12], and sc-GPE [54].

In Table 3, we classify these algorithms according to the Vega-Pons and Ruiz-Shulcloper taxonomy of consensus functions [15]. We also report the single-cell challenges they address [19] (i.e. whether the algorithms generate clustering results with explicit uncertainty values or multiple resolutions), and the availability of their code.

**Table 3.** Overview of scEVE and the seven state-of-the-art scRNA-seq ensemble algorithms surveyed

	Consensus function			Single-cell challenges [19]		Availability	
Method	Median partition [15]	Cells co-occurrences [15]	Clusters robustness	Uncertainty	Resolutions	Code	Reference
EC-PGMGR	.	$\checkmark$	.	.	.	$\checkmark$	[52]
GRACE	.	$\checkmark$	.	.	.	.	[53]
**RSEC**	.	$\checkmark$	.	.	$\checkmark$	$\checkmark$	[13]
SAFE	$\checkmark$	.	.	.	.	$\checkmark$	[14]
SAME	$\checkmark$	.	.	.	.	$\checkmark$	[16]
scEFSC	.	$\checkmark$	.	.	.	$\checkmark$	[12]
sc-GPE	.	$\checkmark$	.	.	.	.	[54]
**scEVE**	.	.	$\checkmark$	$\checkmark$	$\checkmark$	$\checkmark$	.

*Note*: Ensemble algorithms addressing a challenge in single-cell data science are indicated in **bold**.

Out of the five algorithms with available code, only EC-PGMGR [52] was implemented in Matlab instead of R. Except for scEFSC [12], the remaining methods (RSEC [13], SAFE [14], and SAME [16]) all predicted automatically the number of clusters in a dataset. Consequently, they were included in our benchmark, and they were run with their default parameters, according to their respective tutorials and source codes. Since the RSEC algorithm assigns a specific label to cells it cannot cluster, we evaluate its performance with and without these cells (RSEC and RSEC*, respectively).

Each clustering analysis presented in this work was performed on a research server equipped with a dual Intel Xeon CPU E5-2695v3 @ 2.3 GHz processor (with one core and up to 128 GB of total memory). As a result, each clustering method was run with a single core, regardless of its compatibility with multicore calculations (e.g. [14, 16, 24]).

## Results

Two different experiments were conducted to introduce and evaluate our algorithm. In our first experiment, we applied scEVE on a glioblastoma scRNA-seq dataset and thoroughly analyzed the results. In our second experiment, we applied scEVE and a series of clustering methods on experimental and synthetic scRNA-seq datasets, to evaluate their performances.

### Analysis of a glioblastoma dataset with scEVE

**Figure 5.**
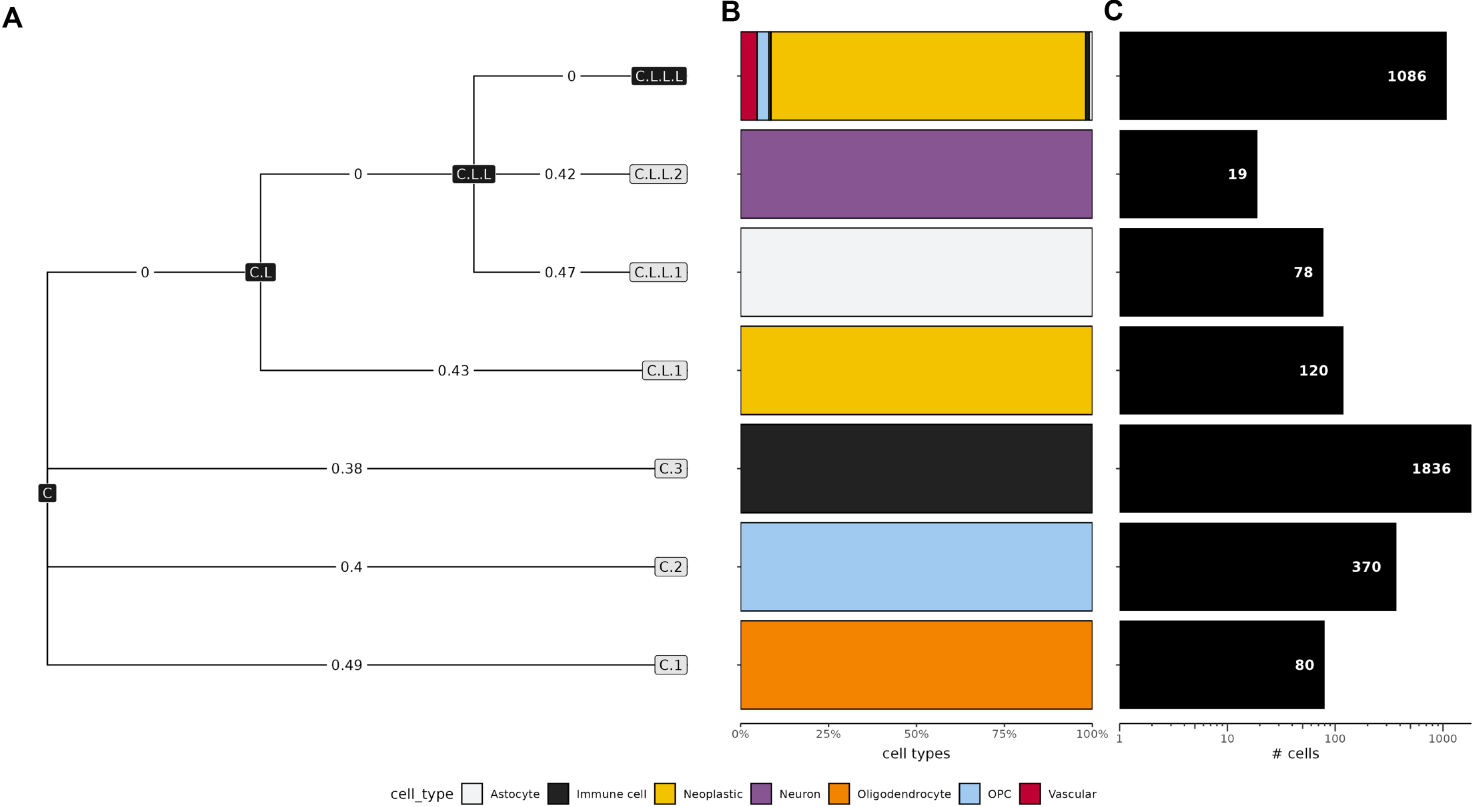
Clustering results of scEVE on the glioblastoma dataset. (**A**) Multi-resolution cluster tree. Clusters predicted by scEVE are encoded as gray and black nodes, for robust and leftover clusters, respectively. Cluster relationships are encoded as edges, and the robustness of a cluster is encoded as an edge weight. (**B**) Barplot of the cell types found in the leaf clusters. The cell types predicted by the authors of the dataset [3] are encoded with colors. Accordingly, homogeneous leaf clusters (i.e. leaf clusters composed of a single cell type) are associated with a monochromatic bar. (**C**) Barplot of the size of the leaf clusters. The exact sizes of the leaf clusters are reported in their respective bars.

At the end of a scEVE clustering analysis, a collection of four spreadsheets is generated: (i) a “meta” spreadsheet indicates the size and robustness of predicted clusters, as well as their hierarchical relationships, (ii) a “samples” spreadsheet reports individual cell composition, (iii) a “features” spreadsheet indicates marker genes and their fold changes, and (iv) a “methods” spreadsheet reports the clustering methods that contributed to each prediction. The meta and samples spreadsheets generated after running our clustering analysis, and the ground truth described by the authors of the dataset [3], were combined to generate Fig. 5. They are also available as Supplementary data.

From this figure, we can see that scEVE was able to predict seven leaf clusters, by recursively subdividing the initial dataset: C.1, C.2, C.3, C.L.1, C.L.L.1, C.L.L.2, and C.L.L.L. Out of these seven clusters, six are robust (i.e. robustness >0.25). They are homogeneous, being composed, respectively, of oligodendrocytes, oligodendrocyte precursor cells (OPCs), immune cells, neoplastic cells (i.e. cancer cells), astrocytes, and neurons. Their size ranges from 19 cells to 1836 cells (0.5% and 51% of the dataset, respectively).

The remaining leftover cluster (C.L.L.L) is hetereogeneous and it includes 1086 cells (30% of the dataset). It comprises a majority of neoplastic cells (89%), as well as some vascular cells (5%), OPCs (3%), and other cell types (3%).

We can see from Fig. 5A that scEVE was able to predict three robust clusters on its first clustering recursion (C.1, C.2, and C.3) and a leftover cluster C.L. From it, scEVE was able to predict a leaf robust cluster C.L.1 and another leftover C.L.L; and from C.L.L, it was able to predict two robust clusters (C.L.L.1 and C.L.L.2), and a leftover cluster C.L.L.L.

**Figure 6.**
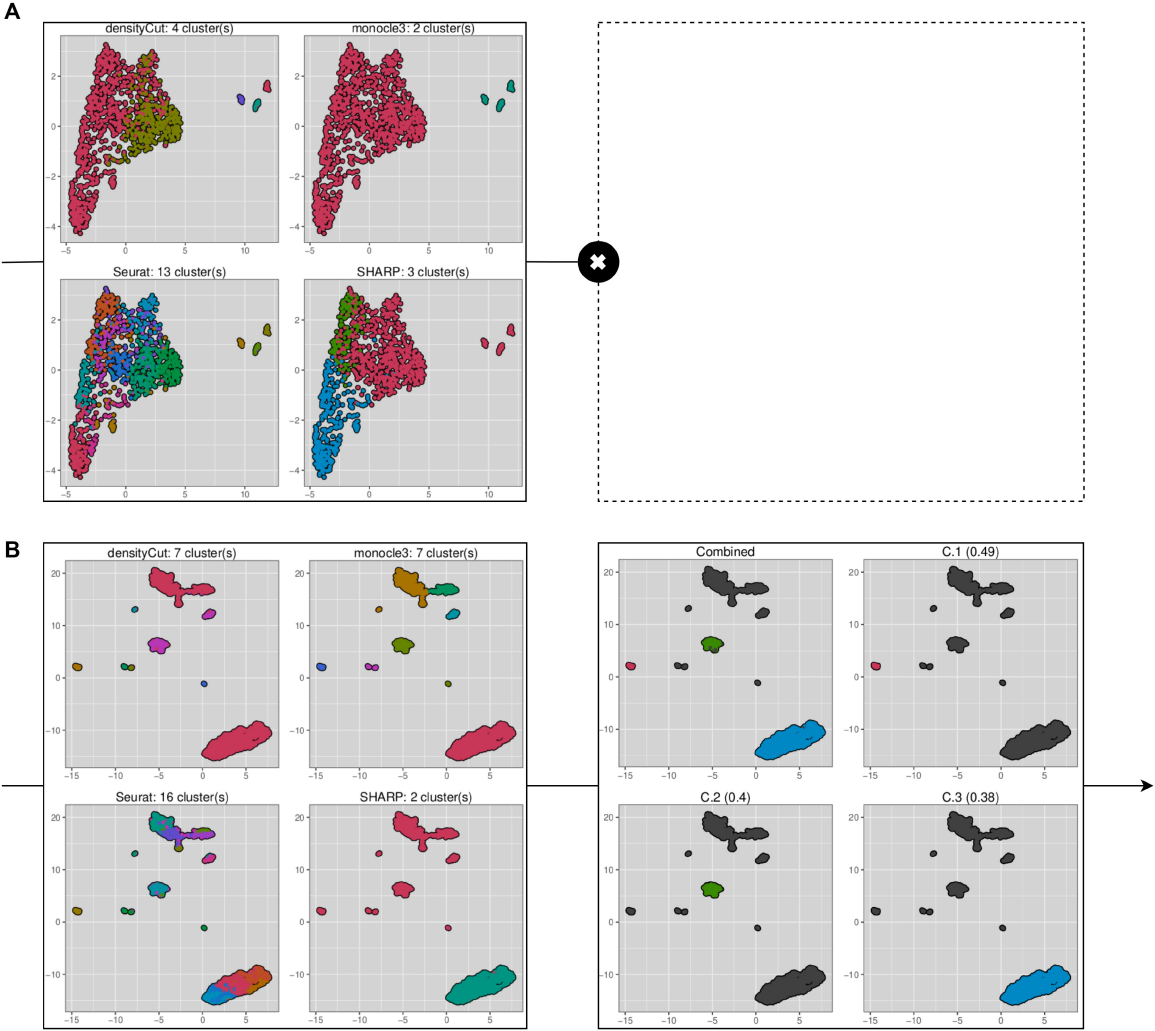
Sample of the descriptive plots automatically generated by scEVE, during the clustering recursions of (**A**) C.L.L.L and (**B**) C. The composite plots on the left side represent base clusters predicted by the methods integrated into scEVE. The composite plots on the right side represent every robust cluster predicted by scEVE. Every subplot is associated with a colored unique robust cluster, and the combined subplot aggregates them together. Cells assigned to the leftover cluster are encoded in black. The absence of right-side plots indicates that no robust clusters were predicted during the clustering recursion. For both left-side and right-side plots, cells are projected on UMAPs [55], and clusters are encoded with color. Colors shared across plots should not be interpreted.

To better understand why scEVE did not subdivide the leaf and leftover cluster C.L.L.L, we investigated the descriptive plots generated automatically during its clustering recursion. A sample of these plots is reported in Fig. 6A. To facilitate this analogy, we also report a sample of the plots generated during the clustering recursion of C (i.e. the initial pool of cells) in Fig. 6B.

The plots show the base and the robust clusters predicted during the recursions. Interestingly, they highlight the considerable impact of the clustering method used on the results of an analysis. Indeed, Fig. 6 shows that the number of predicted clusters—and the boundaries of these clusters—are not consistent from one method to another. Despite these differences, some robust clusters were predicted in C and a descriptive figure representing them was generated (Fig. 6B). The absence of a similar figure for the clustering recursion of C.L.L.L (Fig. 6A) reveals that no robust clusters were identified.

In addition to these sampled plots, descriptive plots are also generated to represent the pools of cells analyzed, and the marker genes of the characterized clusters. Thanks to this property, it is easy to monitor a clustering analysis conducted with scEVE.

#### scEVE detects two distinct cancer cell populations in the glioblastoma dataset

Upon clarifying why the heterogeneous cluster C.L.L.L was not subdivided, we investigated why a homogeneous cluster of cancer cells (i.e. C.L.1) was predicted separately from it (despite the fact that C.L.L.L is teeming with cancer cells). This separation was especially intriguing, given the high robustness of the C.L.1 cluster (*R*_C.L.1_ = 0.43, ranking it third).

According to the “features” spreadsheet of the analysis, 14 and 30 marker genes were detected in the C.L.1 and C.L.L.L clusters, respectively. A manual survey of the GeneCards database [56, 57] and the literature on glioblastoma revealed that both clusters were characterized by some genes involved in cancer (e.g. H19 [58] for C.L.1 and IGFBP2 [59] for C.L.L.L). We employed these 42 marker genes to conduct a comparative Gene Ontology (GO) [60, 61] enrichment analysis. Specifically, we entered them to ToppCluster [62]—a web-based tool that outputs enriched gene annotations from a myriad of knowledge bases—in order to identify GO annotations specific to each cluster. The exhaustive list of GO annotations returned is available as Supplementary data.

Surprisingly, only the “synaptic cleft” GO annotation was returned for C.L.1. On the other hand, 60 annotations were obtained for C.L.L.L. They included molecular functions related to growth factors (e.g. “insulin-like growth factor II binding”), as well as biological processes related to the immune response (e.g. “positive regulation of inflammatory response”) or the angiogenesis (e.g. “fibrinolysis”).

If C.L.L.L was associated with multiple GO annotations related to cancer biology (according to Hanahan and Weinberg cancer hallmarks [63–65]), C.L.1 was not. Faced with that cancer annotation imbalance between the two clusters, we attempted to quantify their cancerous properties with CancerSEA [66], a database that catalogs and classifies genes associated with cancer into functional signatures. For each cluster, we report the number of marker genes shared with a functional signature of cancer in Table 4. To contrast these results, we also indicate the average number of shared genes in a leaf cluster.

**Table 4.** Number of marker genes shared with a functional signature of cancer

	Leaf clusters		
Functional signature	C.L.1 (14)	C.L.L.L (30)	*μ*
Angiogenesis (73)	0	**1**	0.57
Apoptosis (66)	**1**	0	0.14
Cell cycle (137)	0	0	0.14
Differentiation (201)	0	0	0.14
EMT (90)	0	**3**	1
Hypoxia (83)	0	**5**	1
Inflammation (112)	0	1	1.71
Invasion (97)	0	**1**	0.57
Metastasis (166)	0	**4**	1.14
Proliferation (88)	0	**1**	0.57
Quiescence (66)	**1**	0	0.86
Stemness (166)	1	0	1.29
**Any (1244)**	3	**12**	6.57

*Note*: *μ* is the average number of shared genes in a leaf cluster. Values above this average *μ* are encoded in bold.

Up to 12 marker genes were associated with a cancer signature in C.L.L.L (41% of its marker genes), but only three were in C.L.1 (23%). The number and the identity of associated cancer signatures were also different between clusters: C.L.1 was associated with three signatures (apoptosis, quiescence, and stemness), whereas C.L.L.L was associated with seven (angiogenesis, the epithelial-to-mesenchymal transition or EMT, hypoxia, inflammation, invasion, metastasis, and proliferation). Interestingly, the associations of C.L.L.L with EMT, metastasis, and hypoxia are also very strong (ranging from three to five genes, respectively).

Altogether, our extensive analysis of the clusters C.L.1 and C.L.L.L effectively revealed that they were biologically distinct; both were characterized by a different set of genes, and associated with distinct biological processes. Specifically, C.L.1 was associated with synapses and barely associated with any cancer signature, in contrast to C.L.L.L, which was strongly associated with a multitude of cancer signatures. We believe these results are consistent with the existence of a cluster of periphery-tumor cells, and a cluster of core-tumor cells (both sampled by Darmanis *et al.* [3]).

### Evaluation of the performances of the scEVE algorithm

**Figure 7.**
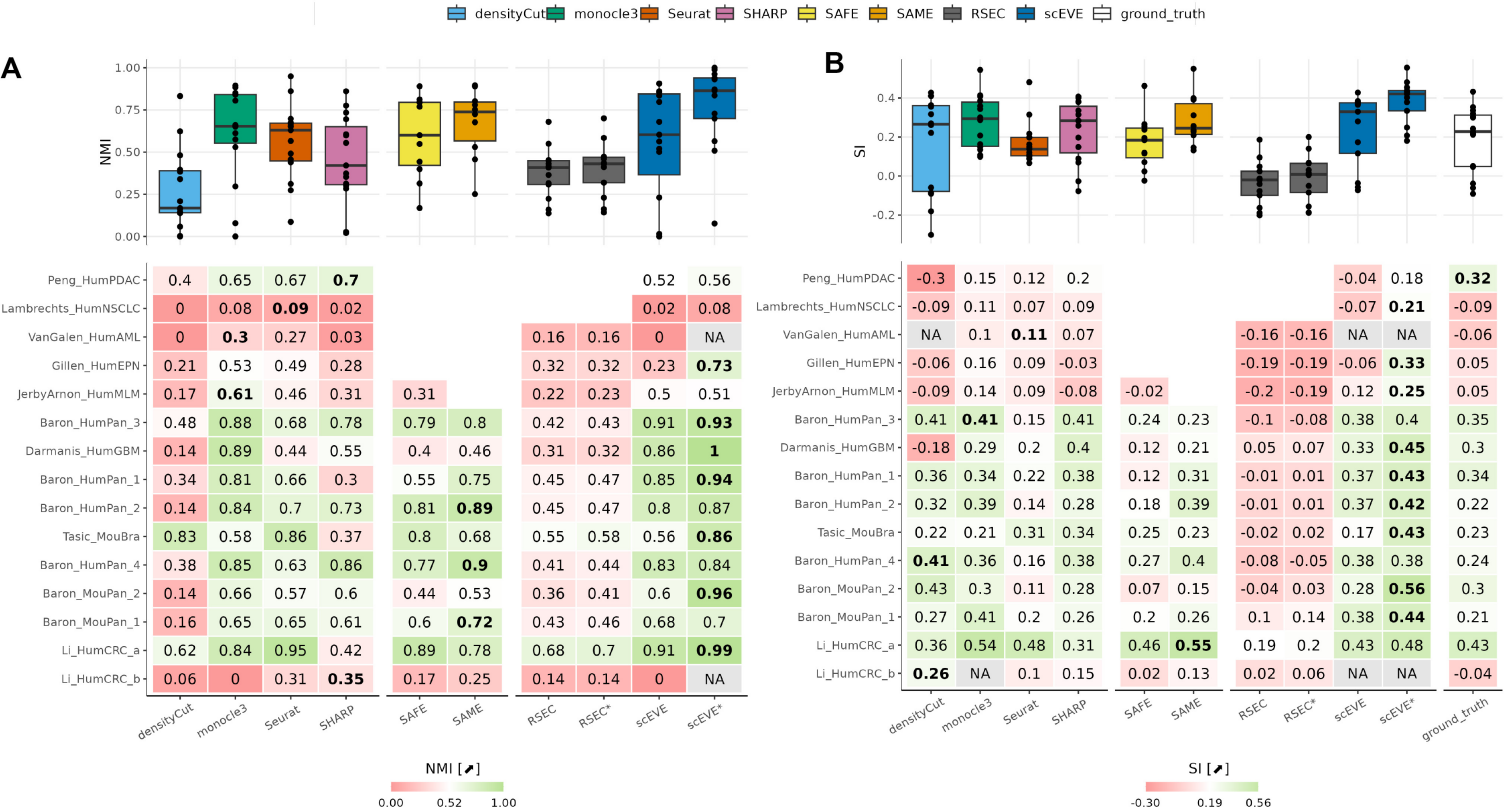
Clustering performances measured on experimental datasets, with two metrics: (**A**) the NMI and (**B**) the SI. Detailed performances are reported in the lower heatmap, and summarized in the upper boxplots. In the lower heatmap, a row is associated with a dataset, a column to an algorithm, and datasets are sorted by descending sizes. The best performance on a dataset is encoded in bold. N/A values indicate that the metric calculation was impossible, and missing values indicate that the clustering analysis was interrupted.

**Figure 8.**
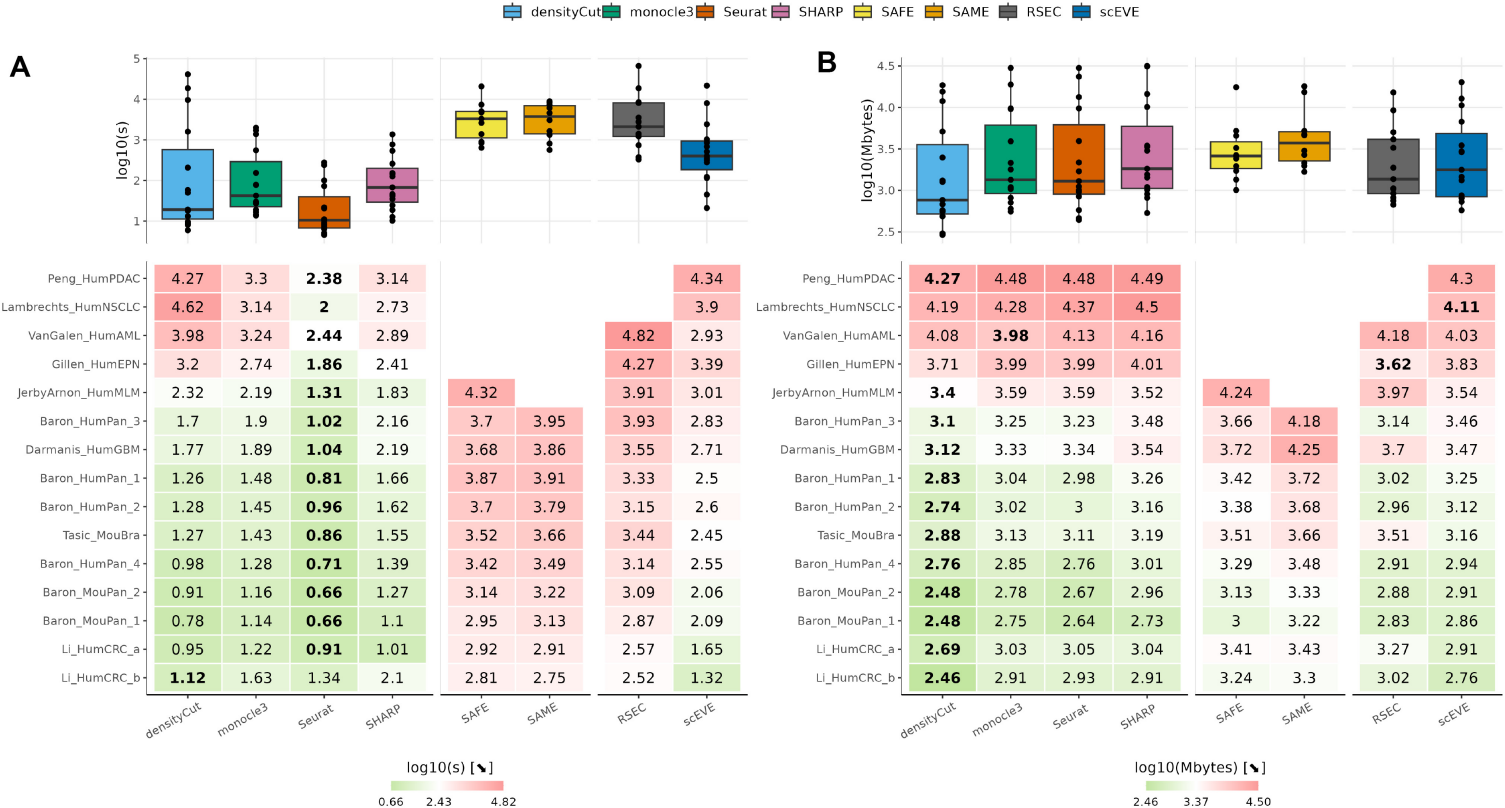
Computational performances measured on experimental datasets, with two metrics: (**A**) the computation time in seconds and (**B**) the peak memory usage in megabytes. Detailed performances are reported in the lower heatmap, and summarized in the upper boxplots. In the lower heatmap, a row is associated with a dataset, a column to an algorithm, and datasets are sorted by descending sizes. The best performance on a dataset is encoded in bold. Missing values indicate that the clustering analysis was interrupted.

We applied the scEVE algorithm on 15 experimental scRNA-seq datasets and compared its performances with the four clustering methods it integrates, and three state-of-the-art scRNA-seq ensemble clustering algorithms. The clustering performance was evaluated using four different metrics. However, for the sake of readability, only the NMI and the SI are reported in this manuscript. The ARI and the nPurity metrics are available as Supplementary data (the conclusions drawn from using either pair of metrics being identical). Figures 7 and 8 summarize the results of our benchmark.

Figure 7A reports the quality of predicted clusters, according to the ground truth established by the authors of the datasets; and it really illustrates how the performances of a clustering algorithm can vary drastically across datasets. For example, monocle3 had the best performance on JerbyArnon_HumMLM (NMI_monocle3_ = 0.61), and the worst on Li_HumCRC_b (NMI_monocle3_ = 0), while SHARP performed best (NMI_SHARP_ = 0.35). This phenomenon is also highlighted by ensemble algorithms: despite being outperformed by the SAME algorithm on most datasets (80%), SAFE performed better than SAME on Li_HumCRC_a (NMI_SAFE_ = 0.89 > NMI_SAME_ = 0.78) and Tasic_MouBra (NMI_SAFE_ = 0.8 > NMI_SAME_ = 0.68).

Three other trends emerge from Fig. 7A: (i) most methods had below-average performances on a subset of five datasets (Lambrechts_HumNSCLC, VanGalen_HumAML, Gillen_HumEPN, JerbyArnon_HumMLM, and Li_HumCRC_b), (ii) densityCut had the lowest performances of all methods, and (iii) RSEC was outperformed by all ensemble algorithms, even when its unassigned cells were filtered (RSEC*).

The performances of our algorithm, scEVE, were above average on all but the five aforementioned datasets. It did not achieve the best ranking on any dataset when the leftover clusters were included in the metric calculation. However, by filtering them out, its performances improved greatly. On some datasets, such as Gillen_HumEPN, the improvement was radical (NMI_scEVE*_ = NMI_scEVE_ + 0.5). As a result, scEVE ranked best on seven datasets (47%), SAME on three (20%), monocle3 and SHARP on two (13%), and Seurat on one (7%). Interestingly, the performance of our algorithm remained below average on Lambrechts_HumNSCLC (NMI_scEVE*_ = 0.08).

Figure 7B reports the cohesion of predicted clusters, with regards to their gene expression. The cohesion of the ground truth proposed by the authors of the datasets is also evaluated. Most of the trends observed in Fig. 7A remain present in Fig. 7B: (i) a method’s performance can vary considerably from one dataset to another, (ii) five datasets are particularly challenging for most methods, and (iii) RSEC is outperformed by all clustering methods. Surprisingly however, densityCut’s performances were drastically better when measured using SI so much so that the method performed best on two datasets: Li_HumCRC_b and Baron_HumPan_4.

Figure 7B also reveals that the five most difficult datasets to process are those with the lowest SI (Lambrechts_HumNSCLC being the lowest). Regardless, for three of them, scEVE was able to predict robust and cohesive clusters (0.21 ≤ SI_scEVE*_ ≤ 0.33). For the other two (VanGalen_HumAML and Li_HumCRC_b), no robust clusters were predicted at all.

The clusters generated by densityCut, monocle3, SHARP, SAME, and scEVE tended to be more cohesive than the clusters of the ground truth. For scEVE, this phenomenon was further exacerbated when the leftover clusters were filtered out; in this case, its robust clusters were always more cohesive than the ground truth, except for Peng_HumPDAC (where the ground truth clusters were the most cohesive, across all predictions).

Overall, with this second clustering metric, scEVE performed best on 9–10 datasets (60%–67%), densityCut on 2 (13%), and monocle3, Seurat, and SAME on 1 (7%). We refer readers interested in a more extensive presentation of our results to the Supplementary data, where figures similar to Fig. 5 are provided for each experimental dataset. Interestingly, the supplementary figures show that the robust clusters predicted by scEVE are often homogeneous (or composed of related cell types), except for Lambrechts_HumNSCLC.

Figure 8A reports the computation times of the algorithms. Predictably, the performances of each algorithm were correlated to the size of the datasets: the smallest datasets were analyzed in a matter of seconds, whereas the largest ones were analyzed after several minutes or hours.

On all but one dataset (93%), Seurat ranked first and was able to predict clusters in 5 min or less. The remaining dataset, Li_HumCRC_b, was analyzed in 16 s by densityCut (instead of 21, for Seurat). Interestingly, densityCut was extremely fast at clustering small datasets, but was outperformed on larger datasets. The performances of monocle3 and SHARP were very similar, and fell between the other two algorithms.

On average, scEVE was 10 times slower than them; but surprisingly, on six datasets, our method outperformed some of them. These contradictory results stemmed from our choice to select a larger number of variable genes, when the clustering methods were run independently (see the “Comparison with the state of the art” section). Clearly, they highlighted a trade-off between exploiting a substantial portion of the transcriptome or keeping the computational cost reasonable.

scEVE was also 10 times faster than the other three ensemble algorithms, on all datasets. In fact, for these algorithms, a single day of computation time was not enough to cluster all datasets. Specifically, SAFE failed to cluster datasets containing more than 18 000 cells (27%), and RSEC failed to cluster datasets containing more than 50 000 cells (13%).

Figure 8B shows the peak memory usage of the algorithms, which is, as expected, correlated with the dataset sizes.

On this metric, densityCut ranked first for 12 datasets (80%), and monocle3, RSEC, and scEVE ranked best for one dataset (7%) each. However overall, the performance differences were small between the methods, with the exception of SAME, which tended to consume an order of magnitude more memory than the other, and consequently, 128 GB of memory was insufficient to perform a SAME clustering analysis on five of our datasets (33%).

#### The properties of robust clusters can vary across datasets

Our evaluation of clustering performances showed that scEVE ranked best when its leftover clusters were filtered. To further assess the impact of this filtering, we calculated the proportion of cells predicted in robust clusters, for each dataset. For comparison, the proportions of cells assigned to clusters by RSEC were also calculated, and the results are presented in Table 5. On average, 67% of the cells across all datasets were assigned to robust clusters with scEVE, while 94% of cells were assigned to clusters with RSEC. Cell proportions varied considerably across datasets, ranging from 99.77% to 13.43%. This variation was even observed in datasets generated by the same authors (e.g. with the Baron *et al.* datasets [2], proportions ranged from 60.62% of the dataset to 99.77%). Remarkably, the three lowest proportions were observed on Lambrechts_HumNSCLC (13.43%), Gillen_HumEPN (29.08%), and Peng_HumPDAC (41.66%), three datasets generated with a 10x Genomics protocol and composed of tens of thousands of cells (Table 2). For each remaining dataset, except Tasic_MouBra (49.85%), the majority of cells were assigned to robust clusters.

**Table 5.** Proportions of cells assigned to clusters

Dataset	RSEC*	scEVE*
Peng_HumPDAC	.	**41.66**
Lambrechts_HumNSCLC	.	**13.43**
VanGalen_HumAML	**97.55**	.
Gillen_HumEPN	**98.85**	29.08
JerbyArnon_HumMLM	**96.73**	64.36
Baron_HumPan_3	96.42	**97.23**
Darmanis_HumGBM	**95.85**	69.74
Baron_HumPan_1	**95.92**	82.6
Baron_HumPan_2	**96**	88.63
Tasic_MouBra	**92.85**	49.85
Baron_HumPan_4	93.71	**99.77**
Baron_MouPan_2	**85.71**	60.62
Baron_MouPan_1	**92.94**	92.58
Li_HumCRC_a	**97.68**	83.6
Li_HumCRC_b	**86.81**	.
**Mean ± SE**	**94.39 ± 1.04**	67.16 ± 7.04

*Note*: Highest proportions are encoded in bold.

By exploiting the “methods” spreadsheets of every analysis, we were also able to investigate which base methods were contributing to the prediction of robust clusters. These results are presented in Table 6. Strikingly, for each dataset except Peng_HumPDAC, 100% of the detected robust clusters were associated with a monocle3 base cluster (for an average participation of 99.38%). densityCut and Seurat were also two major contributors in detecting robust clusters; on average, 92.53% and 86.18% of the robust clusters in a dataset were associated with one of their base clusters. Remarkably, Seurat’s participation was the highest (100%) on datasets generated with 10x Genomics or SMARTER protocols (Table 2). Finally, SHARP played a minor role in detecting robust clusters (44.84%), which varied drastically across datasets, ranging from 0% (on Gillen_HumEPN and Tasic_MouBra) to 100% (on Baron_HumPan_4 and Li_HUMCRC_a).

**Table 6.** Contributions of the clustering methods in detecting robust clusters

Dataset	densityCut	monocle3	Seurat	SHARP
Peng_HumPDAC	96	92	**100**	24
Lamb_HumNSCLC	**100**	**100**	**100**	11.11
Gill_HumEPN	**100**	**100**	**100**	0
Jerb_HumMLM	**100**	**100**	87.5	25
Bar_HumPan_3	88.89	**100**	66.67	88.89
Darm_HumGBM	66.67	**100**	83.33	50
Bar_HumPan_1	**100**	**100**	88.89	22.22
Bar_HumPan_2	88.89	**100**	88.89	66.67
Tas_MouBra	**100**	**100**	**100**	0
Bar_HumPan_4	62.5	**100**	75	**100**
Bar_MouPan_2	**100**	**100**	80	20
Bar_MouPan_1	**100**	**100**	50	75
Li_HumCRC_a	**100**	**100**	**100**	**100**
**Mean**	92.53	**99.38**	86.18	44.84
**± SE**	± 3.63	± **0.62**	± 4.26	± 10.3

*Note*: The contribution of a method corresponds to the percentage of robust clusters exploiting one of its base clusters. Highest contributions are encoded in bold.

#### scEVE clusters unrelated cell populations successfully, according to simulations

Using synthetic datasets, we investigated which data properties could influence the performances of our algorithm. Specifically, we evaluated it on up to 1200 synthetic datasets, with different numbers of clusters, cluster sizes, and cluster transcriptomes (see the “Generation of synthetic scRNA-seq datasets” section). The clustering methods integrated into scEVE were also evaluated.

**Figure 9.**
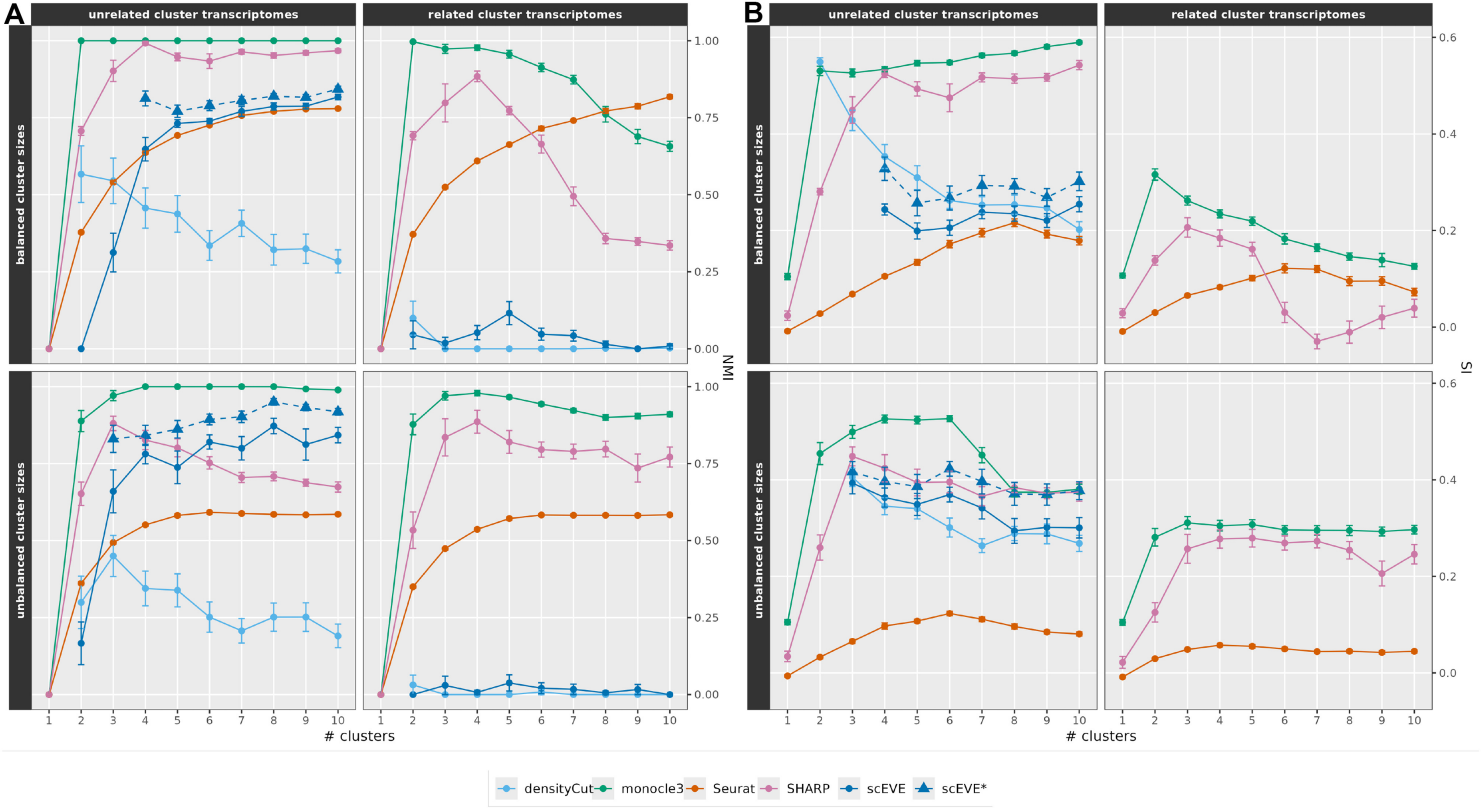
Clustering performances measured on synthetic datasets according to two metrics: (**A**) the NMI and (**B**) the SI. The mean performances are encoded with lines and points, and the standard errors are encoded with error bars. The performance of scEVE without its leftover clusters (scEVE*) is encoded with a dashed line. The calculation of the NMI yields no results when two datasets composed of a single cluster are compared. Similarly, the calculation of the SI yields no results when a set composed of a single cluster is evaluated (Supplementary data). To account for these properties, only the experiments for which a majority of the replicates (*n* > 15) yielded results are reported.

Figure 9 summarizes the clustering performances measured with the NMI (Fig. 9A) and the SI (Fig. 9B). The ARI and the nPurity, as well as the computational performances of the methods, are all available as Supplementary data.

Figure 9A shows that monocle3 performed best on every data configuration and was able to perfectly predict clusters in unrelated datasets. SHARP was second on two configurations (balanced unrelated datasets, and unbalanced related datasets), and Seurat and scEVE were second on the remaining two configurations (balanced related datasets, and unrelated unbalanced datasets, respectively). Finally, for every configuration, densityCut was outperformed. Once again, scEVE’s performances were improved by filtering out the leftover clusters (scEVE*). On the flip side, it was also the only method, along with densityCut, that did not over-cluster datasets composed of a single cell population (Supplementary data). On the downside, our method struggled to cluster datasets composed of two- to three-cell populations. The performances of all methods, except Seurat, tended to decrease when the cluster transcriptomes were related. This was exacerbated for densityCut and scEVE, whose performances collapsed close to zero. Manual inspection of the descriptive plots generated during the analyses of these datasets revealed that scEVE was in fact able to predict robust clusters; however, they were biologically too similar and were therefore filtered out. Only in rare cases was a single robust cluster predicted.

When the SI was used instead of the NMI (Fig. 9B), two main differences emerged: (i) densityCut outperformed Seurat on unrelated datasets and (ii) both densityCut and scEVE were missing in the right column of the plot. The absence of the two methods was in fact consistent with the poor performances reported in Figure 9A: when cluster transcriptomes were highly related, both methods were unable to predict clusters, and the calculation of the SI yielded no results (Supplementary data).

## Discussion

In the past decade, scRNA-seq analyses have been established as a pivotal tool for modern biology [4–6]. Accordingly, hundreds of computational methods are developed to carry out the crucial step of cell clustering [7, 8]. Because of this methodological surge, the analysis of a single dataset can now yield hundreds of contradictory results.

Ensemble algorithms integrate multiple methods and minimize the differences of their predictions to predict clusters that are robust to the method used [15]. However, these algorithms still face some conceptual challenges in single-cell data science [19]: they do not generate clustering results with uncertainty values and multiple resolutions.

In this work, we attempted to tackle these challenges, by proposing an alternative approach to the ensemble clustering problem. Specifically, we hypothesized that prediction differences between clustering methods were an informative signal to be described, rather than a methodological bias to be minimized. To test our hypothesis, we developed scEVE, a recursive ensemble algorithm that embraces this philosophy. In this work, two different experiments were conducted to present and to evaluate our algorithm.

In the first experiment, a gliobastoma dataset [3] was analysed with scEVE. In doing so, we were able to show that scEVE efficiently generates clustering results with uncertainty values and multiple resolutions (Fig. 5). Our results also highlighted that scEVE is a transparent algorithm, which documents each clustering recursion with a set of descriptive plots (Fig. 6). Thanks to this combination of properties (and by leveraging multiple knowledge bases [61, 66]), we were able to reveal the existence of two biologically distinct subclusters of cancer cells in the dataset (Table 4).

In the second experiment, extensive benchmarks were conducted to evaluate scEVE, the four clustering methods integrated into it (Table 1), and three state-of-the-art scRNA-seq ensemble algorithms [13, 14, 16].

A benchmark was based on 15 public experimental scRNA-seq datasets (Table 2), and showed that our algorithm had great clustering performances, which were further improved when the uncertainties it quantified were taken into account (Fig. 7). It also revealed that scEVE is a frugal ensemble algorithm, compared to the state of the art (Fig. 8). Finally, it also highlighted the existence of a trade-off in single-cell analyses, between leveraging all cells of a dataset and the risk of analyzing ambiguous clusters, or analyzing certified clusters only, and ignoring part of the dataset (Table 5). We believe that this trade-off can be approached in both directions, depending on the motivations of the experimenters.

A second benchmark was conducted, with up to 1200 synthetic datasets. These were generated using a data simulator [43] and by altering cluster properties to emulate different biological scenarios (Fig. 4). Using this novel approach, we found that scEVE would perform well on datasets with unrelated cell types (Fig. 9), but would struggle on datasets where cell types are highly related.

Interestingly, the results of our experimental benchmark confirmed the findings of Yu *et al.* [11] when the NMI metric [17] was used: monocle3 [21] and Seurat [23] performed best, while densityCut [22] and SHARP [24] performed worst. However, when the SI metric was used [45], densityCut, SHARP, and monocle3 all performed as well as or even better than Seurat. In our synthetic benchmark, SHARP was the second best method regardless of the metric used, and densityCut was the only method able to prevent over-clustering. Overall, these results highlight the need to use multiple metrics and multiple methods in single-cell data science. They also raise interrogations regarding the quality of the datasets used in our field (Fig. 7B).

scEVE integrates four different clustering methods. This number is similar to that of other ensemble algorithms (e.g. [14, 16, 52]), but the integrated methods may differ. In our work, we employed methods ranked from best to worst (according to [11]), and we were still able to demonstrate the great performance of scEVE. Certainly, exploring the impact of its hyper parameters and fine-tuning them would further improve its performances. In our evaluation, feature selections were performed according to best practices [6] or authors’ recommendations, but exploring the impact of different selection strategies on ensemble algorithms could also provide interesting insights.

As a whole, the problem of downstream biological analyses in single-cell data science is intricate. First, it depends on clustering results, which are inconsistent between methods. In our work, we have illustrated this inconsistency multiple times. In the literature, the existence of benchmark studies (e.g. [9–11]) exemplifies these inconsistencies. It has also been shown that the performance of a single method is inconsistent across datasets, due to its sensitivity to hyper parameters [10] and specific data settings [9, 11]. Our own results support this observation, and emphasize the importance of using ensemble methods.

Because of this inherent irregularity, cell populations predicted by clustering analysis must always be verified. However, given the substantial cost of experimental verification (both in terms of time and money [67]), the field of single-cell data science would benefit greatly from the development of earlier computational verification approaches.

One such approach to verify clusters computationally consists of exploiting their marker genes (e.g. [2, 3, 40]). Intuitively, if all clusters express marker genes specific to a different cell type—according to some prior knowledge (e.g. [68–70])—their prediction is compatible with our current understanding of biology, and they are probably valid. However, by leveraging only a handful of known marker genes, this approach can dismiss novel cell populations and new biological insights [71].

In cancer specifically, where classification based on cell type is fundamentally fuzzy [72, 73], this approach might be insufficient. Our results illustrated this limitation: in the first experiment, the two cancer cell populations detected by scEVE were characterized by EGFR, a known glioblastoma marker gene [74]. However, they were still biologically distinct, and we revealed this by employing 44 marker genes.

Another approach to verifying clusters seen in the literature (e.g. [37]) is to project a nonlinear dimensionality reduction of the data onto two dimensions (e.g. using t-SNE [75] or UMAP [55]), and to compare the shapes drawn on the visualization with the predicted clusters. However, given the limitations of these visualizations, such as their sensitivity to hyper parameters [76, 77], this second approach is also unwise.

In this work, we argue that quantifying the uncertainty of clusters—across multiple resolutions—can effectively help verify their existence, and unravel new biological insights. For instance, the uncertainties measured could help in prioritizing the follow-up experiments to conduct, and it could also help in discovering new cell types. In our own experiment, we were motivated to investigate the biology of a cancer cell cluster thanks to its high robustness, and we revealed that its biology was different from the other cancer cells of the sample.

We also argue that conducting transparent clustering analyses facilitates this verification; in the first experiment again, understanding why certain clusters of cells were not further divided was very straightforward, thanks to scEVE documenting each of its recursions.

Altogether, our work reveals that scEVE is the first ensemble clustering algorithm that addresses multiple conceptual challenges in single-cell data science [19]. We also showed that scEVE outperforms state-of-the-art methods and that its predictions are designed to prevent over-clustering. For all these reasons, we believe that future analyses in single-cell data science will benefit from the use of our algorithm, and that method developers will take advantage of the novel philosophy we propose to address the ensemble clustering problem.

## Supplementary Material

lqaf073_Supplemental_Files

## Data Availability

The scEVE algorithm is an instance of fEVE, our open framework for -omics ensemble clustering analyses. fEVE is implemented in an R package and is available in the following GitHub repository: https://github.com/yanisaspic/fEVE. The codes used to generate the datasets and the results presented in this paper are available in the following public Figshare repository: 10.6084/m9.figshare.28804547.v1.
