# Supplementary material for: scEVE: a single-cell RNA-seq ensemble clustering algorithm capitalizing on the differences of predictions between multiple clustering methods
Source: NAR Genom Bioinform. 2025 Jun 9;7(2):lqaf073. doi: 10.1093/nargab/lqaf073 (PMC12147100; doi:10.1093/nargab/lqaf073)
Supplement: lqaf073_Supplemental_Files [file lqaf073_supplemental_files.zip › Supplementary_Data.pdf]

Supplementary Material

Equations of the clustering metrics.

The `aricode` library [1] calculates the Adjusted Rand Index (ARI) [2] and the Normalized Mutual Information (NMI) [3] with **Supplementary Equation 1** and **Supplementary Equation 3**, respectively. The equations are described in Vinh *et al.* [4], and we contextualize them in this appendix. We use the `bluster` library [5] to calculate the Silhouette Index (SI), and we contextualize it in **Supplementary Equation 6**.

$$ARI(U,V) = \frac{\sum_{i,j}\binom{N_{u_i \cap v_j}}{2} - EI(U,V)}{\frac{1}{2}\left[\sum_i \binom{N_{u_i}}{2} + \sum_j \binom{N_{v_j}}{2}\right] - EI(U,V)} \quad (1)$$

where,
$ARI(U,V)$ is the Rand Index of the clustering results U and V adjusted for chance,
N_{u_i} is the number of cells in the cluster i, from the clustering results U,
$N_{u_i \cap v_j}$ is the number of cells in both clusters i and j, from the clustering results U and V, respectively, and
$EI(U,V)$ is described in **Supplementary Equation 2**,

$$EI(U,V) = \frac{\sum_i \binom{N_{u_i}}{2} \sum_j \binom{N_{v_j}}{2}}{\binom{N}{2}} \quad (2)$$

where,
$EI(U,V)$ is the Rand Index of the clustering results U and V expected by chance, and
N is the total number of cells.

$$NMI(U,V) = \frac{I(U,V)}{\max(H(U),H(V))} \quad (3)$$

where,
$NMI(U,V)$ is the max-normalized Mutual Information of the clustering results U and V,
$I(U,V)$ is described in **Supplementary Equation 4**, and
$H(U)$ is described in **Equation 5**.

$$I(U,V) = \sum_i \sum_j p(u_i,v_j) \log \frac{p(u_i,v_j)}{p(u_i)p(v_j)} \quad (4)$$

where,
$I(U,V)$ is the mutual information between the clustering results U and V, and
$p(u_i,v_j)$ is the proportion of cells in both clusters i and j, from the clustering results U and V, respectively.

$$H(U) = -\sum_i p(u_i) \log p(u_i) \quad (5)$$

where,
$H(U)$ is the entropy of the clustering results U, and
$p(u_i)$ is the proportion of cells in the cluster i, from the clustering results U.

$$SI_{c^k} = \frac{d_{out}(c^k) - d_{in}(c^k)}{max(d_{in}(c^k), d_{out}(c^k))} \quad (6)$$

where,
SI_{c^k} is the Silhouette Index of a cell c predicted in a cluster k,
$d_{out}(c^k)$ is the average distance between this cell and the cells predicted outside of the cluster k, and
$d_{in}(c^k)$ is the average distance between this cell and the cells predicted inside of the clutster k.

Note that $NMI(U,V)$ is undefined if $H(U) = 0$ and $H(V) = 0$, *i.e.* if the results of the clustering analysis and the ground truth both comprise a unique cluster. Also note that SI_{c^k} is undefined if $d_{out}(c^k)$ or $d_{in}(c^k)$ are missing, *i.e.* if a single cluster is predicted in the dataset.

Regarding our evaluation of the clustering methods, the Silhouette Index (SI) and the neighbor Purity (nPurity) were calculated using 20 principal components, generated from the 5,000 most variable genes of each dataset (according to the tutorial of the `bluster` package).

Errors raised during our experiments.

Our attempts at generating base clusters with the functions readily implemented in the `SAFE` and `SAME` packages yielded errors. We believe these errors originated from a modification of the `SC3` package, or one of its dependencies. Because of that, we re-implemented these functions ourselves, according to the source codes of the two packages. In order to run the SAFE algorithm on our server, we also had to develop a virtual environment that included every 32-bit Ubuntu dependency required to run the `shmetis` program. Finally, when parallel instances of the SAFE and SAME algorithms were run on our server simultaneously, the analyses would be interrupted by errors. We were not able to identify the origin of these errors. Accordingly, we had to run every clustering analysis using the SAFE or SAME algorithms, sequentially. We also noted that the results of SAFE and SAME were a little variable across replicates, despite defining a random state explicitly, and leveraging the same exact base clusters.

We also noted that the analysis of some synthetic replicates would yield errors. These errors were not specific to a data configuration or a random state. We were not able to identify their origin, and to account for them, only results observed on a majority of replicates ($n > 15$) were reported in our work.

Supplementary Figures

Our evaluation of the clustering methods' performances on experimental datasets, with the ARI and the nPurity metrics, is reported in **Supplementary Figure 1**. Our evaluation with the same metrics, on the synthetic datasets, is reported in **Supplementary Figure 2**. Finally, our evaluation of their computation times and peak memory usages, on these synthetic datasets, is reported in **Supplementary Figure 3**.

The meta and samples spreadsheets generated after running our clustering analyses on experimental datasets, and the ground truth described by the authors of these datasets, were combined to generate **Supplementary Figures 4 to 15**. Because every figure shares the same description as **Supplementary Figure 4**, we do not describe **Supplementary Figures 5 to 15**, so as to reduce the size and to improve the readability of our Supplementary Figures.

Our remaining Supplementary Data, namely (i) the spreadsheets generated during our clustering analysis of the glioblastoma dataset, and (ii) the exhaustive list of GO annotations returned by ToppCluster during our comparative analysis of the two cancer clusters, are all available for download at NAR Online.

References

1. Julien Chiquet, Guillem Rigaill, and Martina Sundqvist. *aricode: Efficient Computations of Standard Clustering Comparison Measures*, 2023.
2. Lawrence Hubert and Phipps Arabie. Comparing partitions. *Journal of Classification*, 2(1):193–218, 1985.
3. Alexander Strehl and Joydeep Ghosh. Cluster Ensembles - A Knowledge Reuse Framework for Combining Multiple Partitions. *Journal of Machine Learning Research*, 3:583–617, 2002.
4. Nguyen Xuan Vinh, Julien Epps, and James Bailey. Information theoretic measures for clusterings comparison: is a correction for chance necessary? In *Proceedings of the 26th Annual International Conference on Machine Learning*, ICML '09, pages 1073–1080, 2009.
5. Aaron Lun. *bluster: Clustering Algorithms for Bioconductor*, 2023.

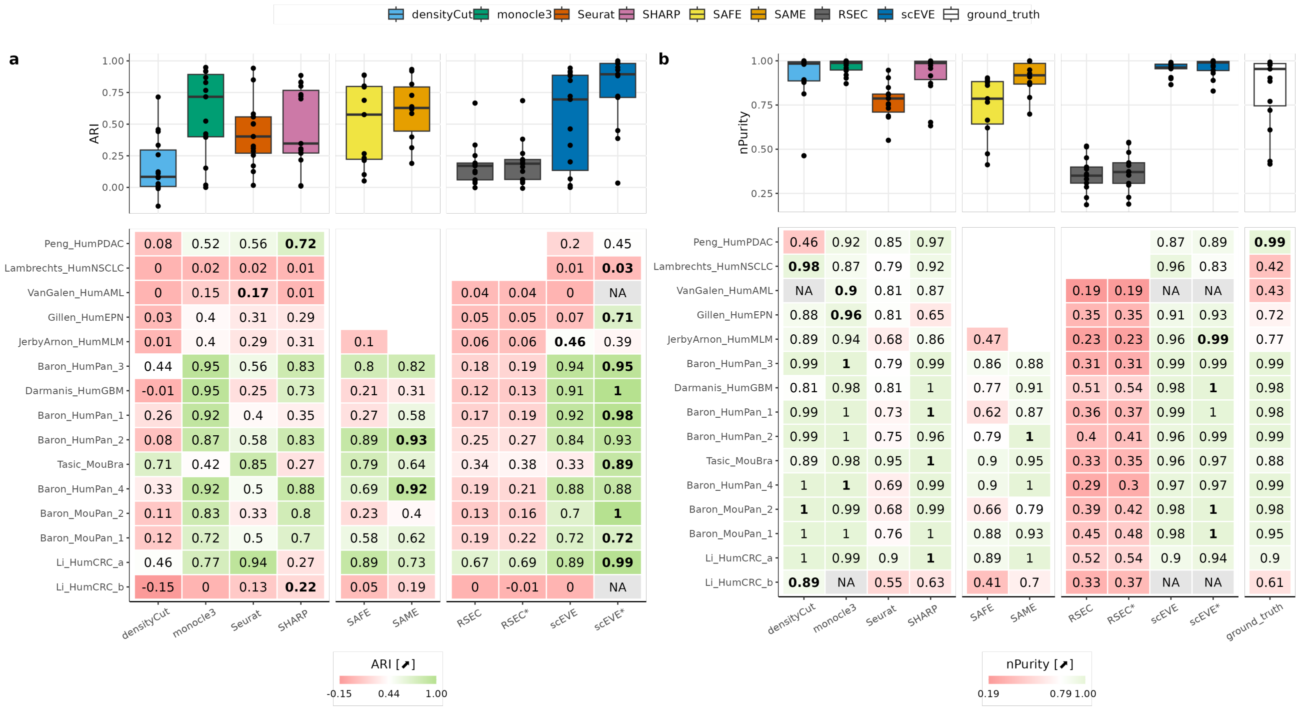

Supplementary Fig. 1. Clustering performances measured on experimental datasets, with two metrics: (a) the ARI, and (b) the nPurity. Detailed performances are reported in the lower heatmap, and summarized in the upper boxplots. In the lower heatmap, a row is associated to a dataset, a column to an algorithm, and datasets are sorted by descending sizes. The best performance on a dataset is encoded in bold. Performances below- and above- the global average are encoded in red and green, respectively. N/A values indicate that the metric calculation was impossible, and missing values indicate that the clustering analysis was interrupted.

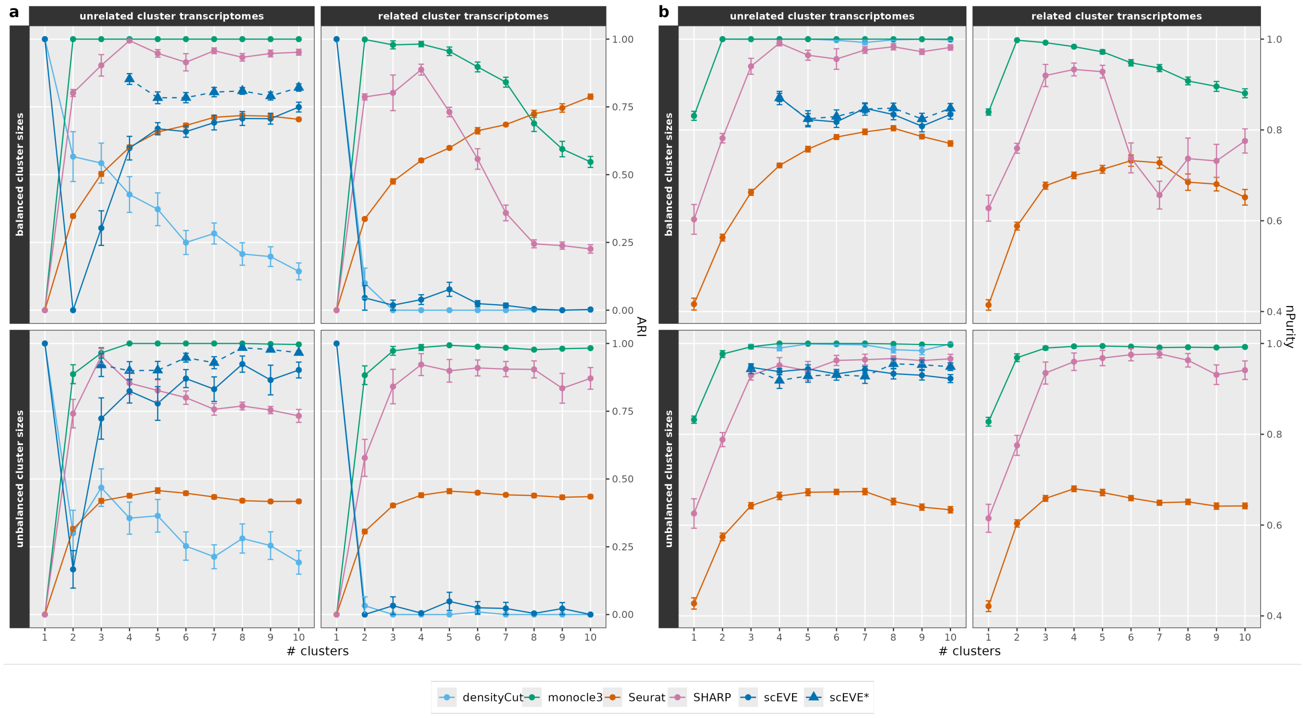

Supplementary Fig. 2. Clustering performances measured on synthetic datasets, with two metrics: (a) the ARI, and (b) the nPurity. The mean performances are encoded with lines and points, and the standard errors are encoded with error bars. The performance of scEVE without its leftover clusters (scEVE*) is encoded with a dashed line. The calculation of the nPurity yields no results when a set composed of a single cluster is evaluated. To account for this property, only the experiments for which a majority of the replicates ($n > 15$) yielded results are reported.

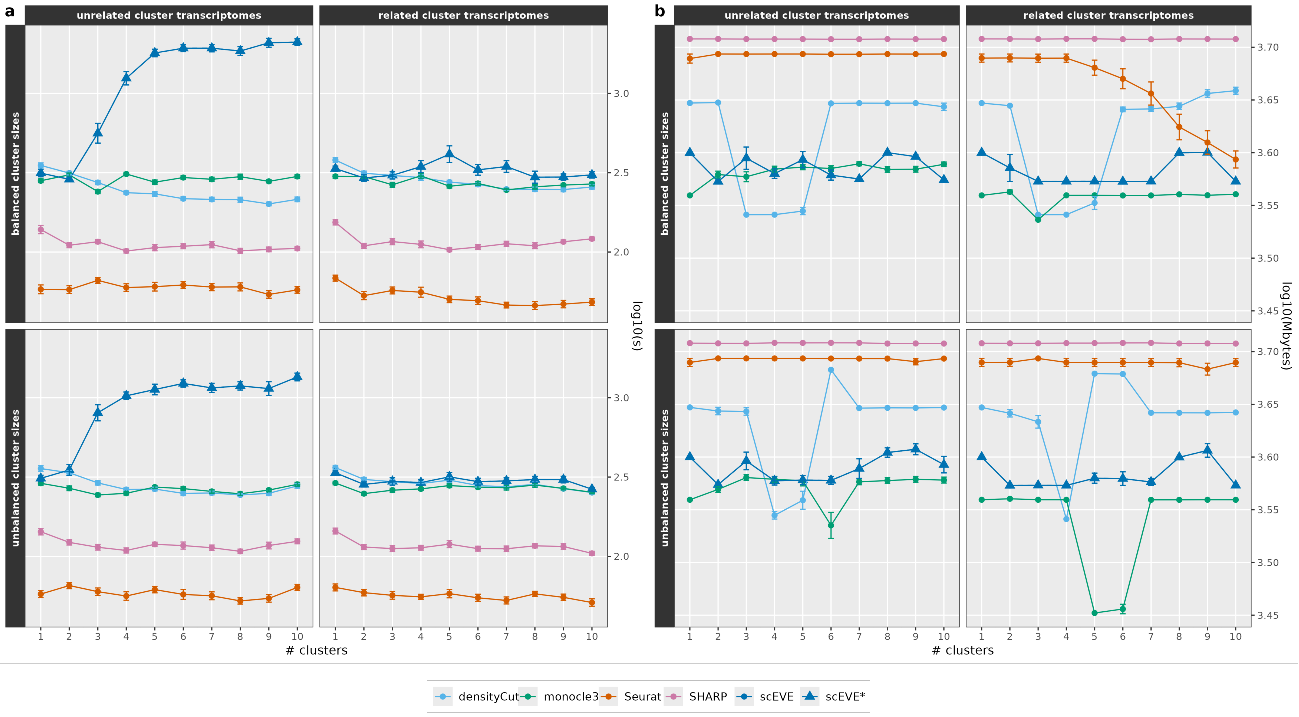

Supplementary Fig. 3. Computational performances measured on synthetic datasets, with two metrics: (a) the computation time in seconds, and (b) the peak memory usage in megabytes.

The mean performances are encoded with lines and points, and the standard errors are encoded with error bars. The performance of scEVE without its leftover clusters (scEVE*) is encoded with a dashed line.

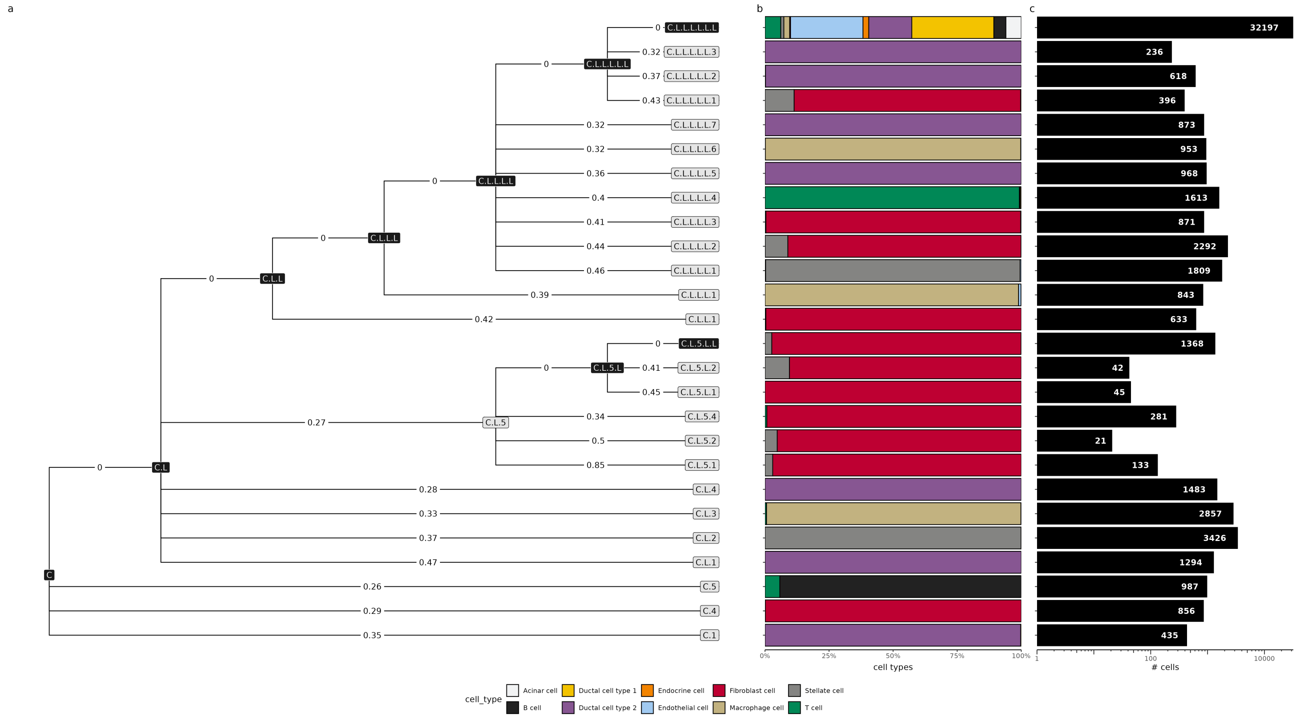

Supplementary Fig. 4. Clustering results of scEVE on the Peng_HumPDAC dataset.
(a) Multi-resolution cluster tree. Clusters predicted by scEVE are encoded as grey and black nodes, for robust and leftover clusters, respectively. Cluster relationships are encoded as edges, and the robustness of a cluster is encoded as an edge weight.
(b) Barplot of the cell types found in the leaf clusters. The cell types predicted by the authors of the dataset are encoded with colors. Accordingly, homogeneous leaf clusters (*i.e.* leaf clusters composed of a single cell type) are associated with a monochromatic bar.
(c) Barplot of the size of the leaf clusters. The exact sizes of the leaf clusters are reported in their respective bars.

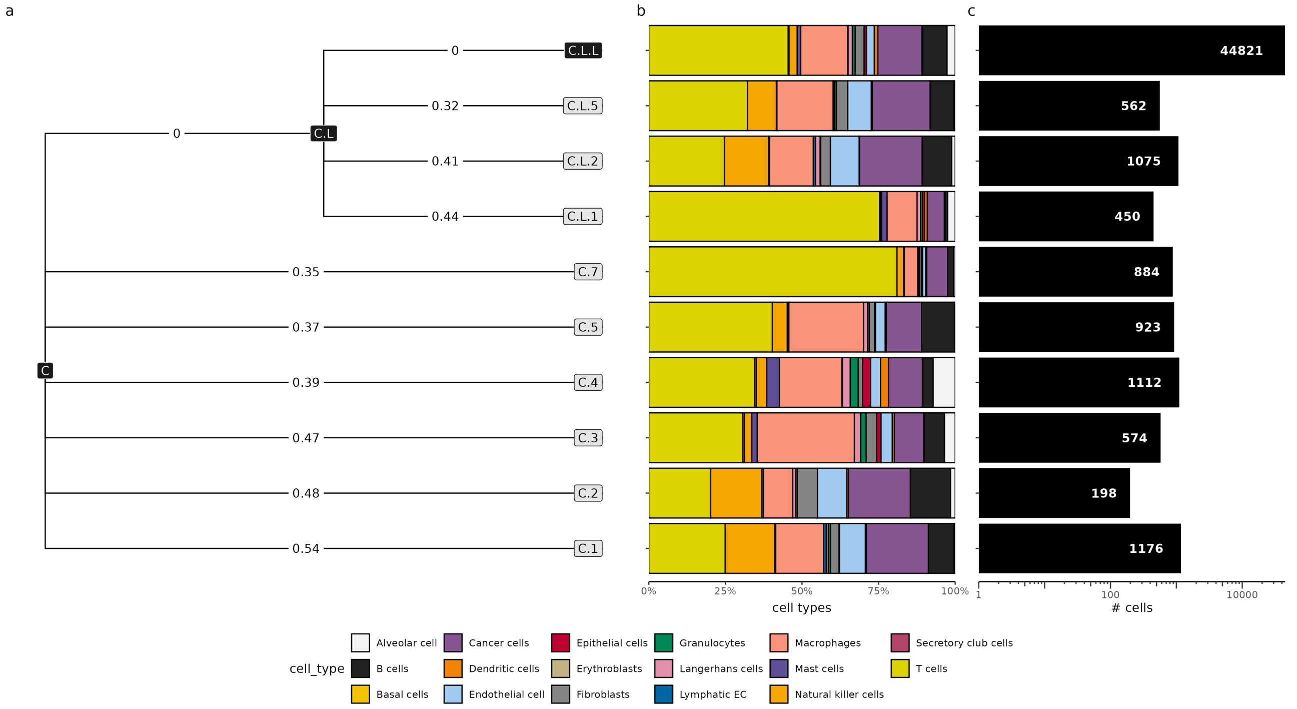

Supplementary Fig. 5. Clustering results of scEVE on the Lambrechts_HumNSCLC dataset.

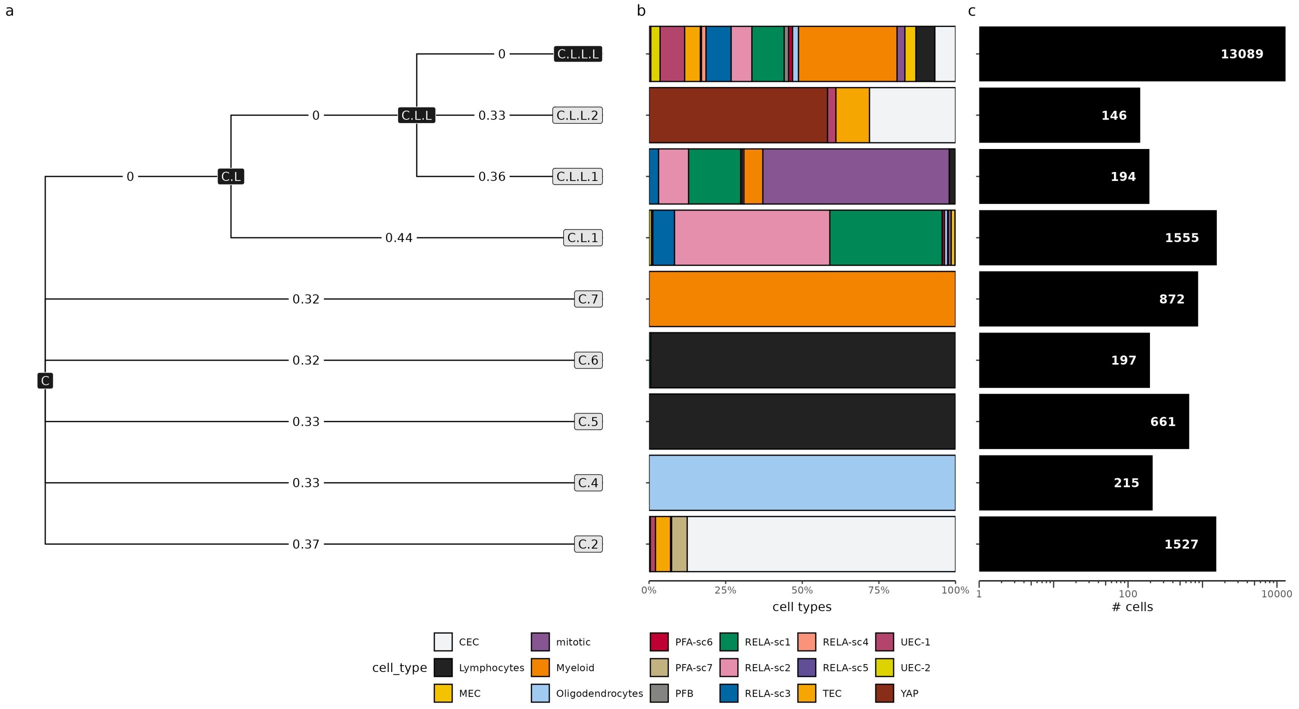

Supplementary Fig. 6. Clustering results of scEVE on the Gillen_HumEPN dataset.

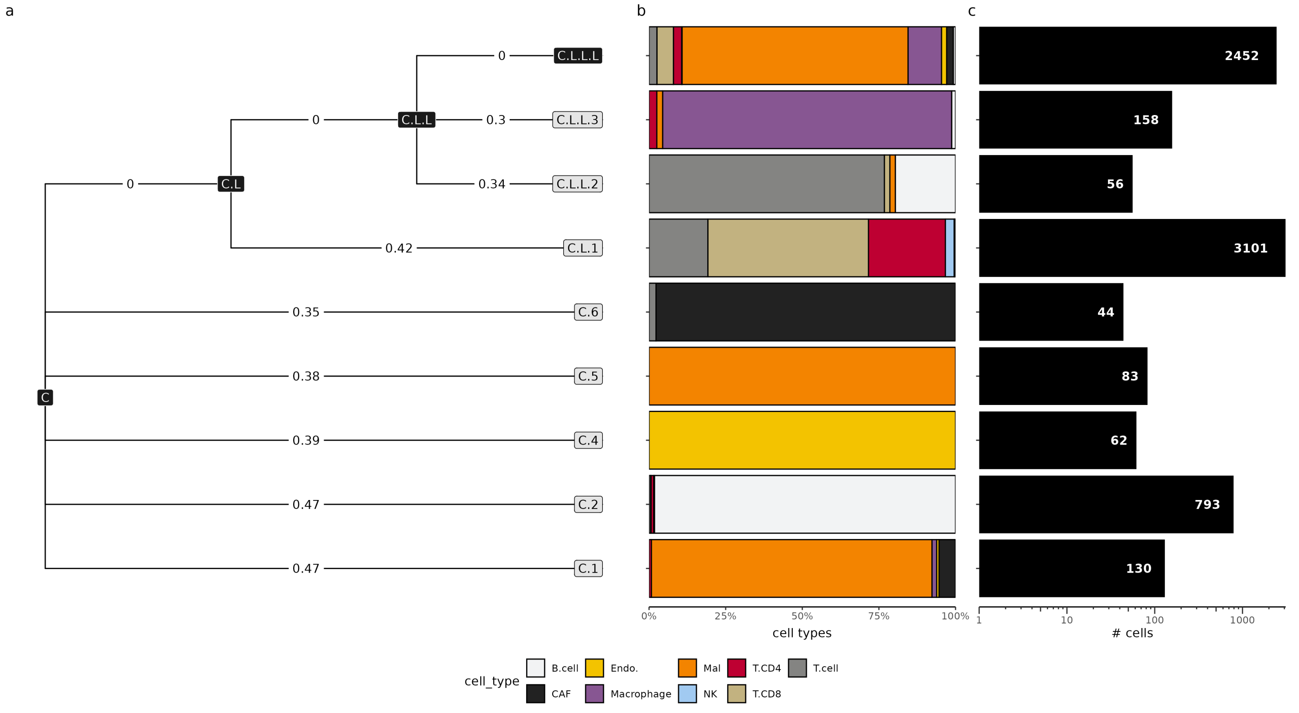

Supplementary Fig. 7. Clustering results of scEVE on the JerbyArnon_HumMLM dataset.

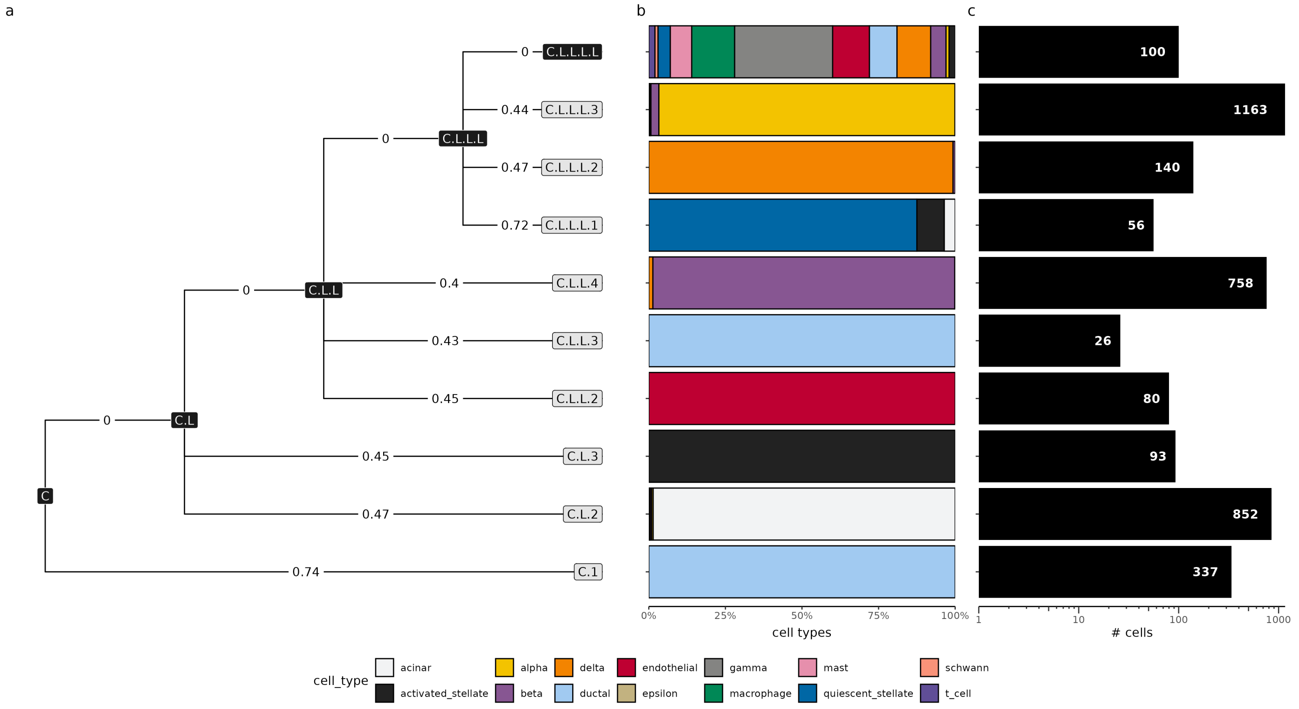

Supplementary Fig. 8. Clustering results of scEVE on the Baron_HumPan_3 dataset.

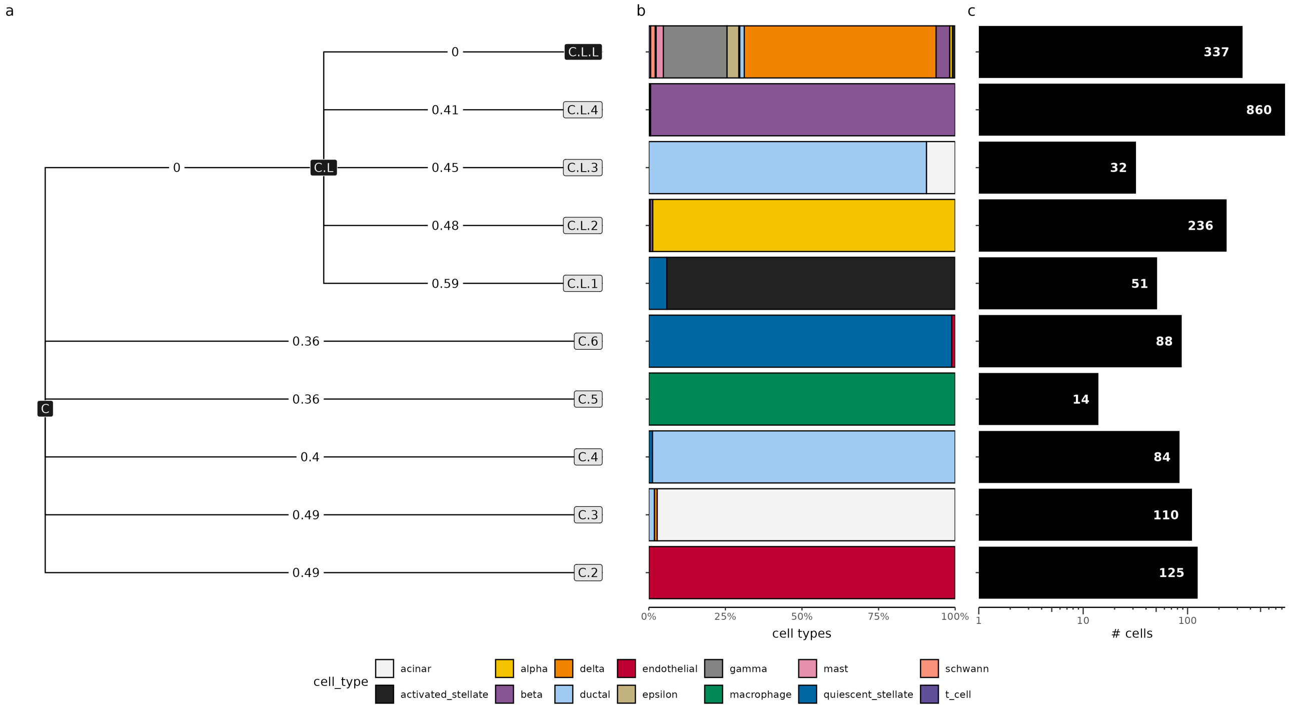

Supplementary Fig. 9. Clustering results of scEVE on the Baron_HumPan_1 dataset.

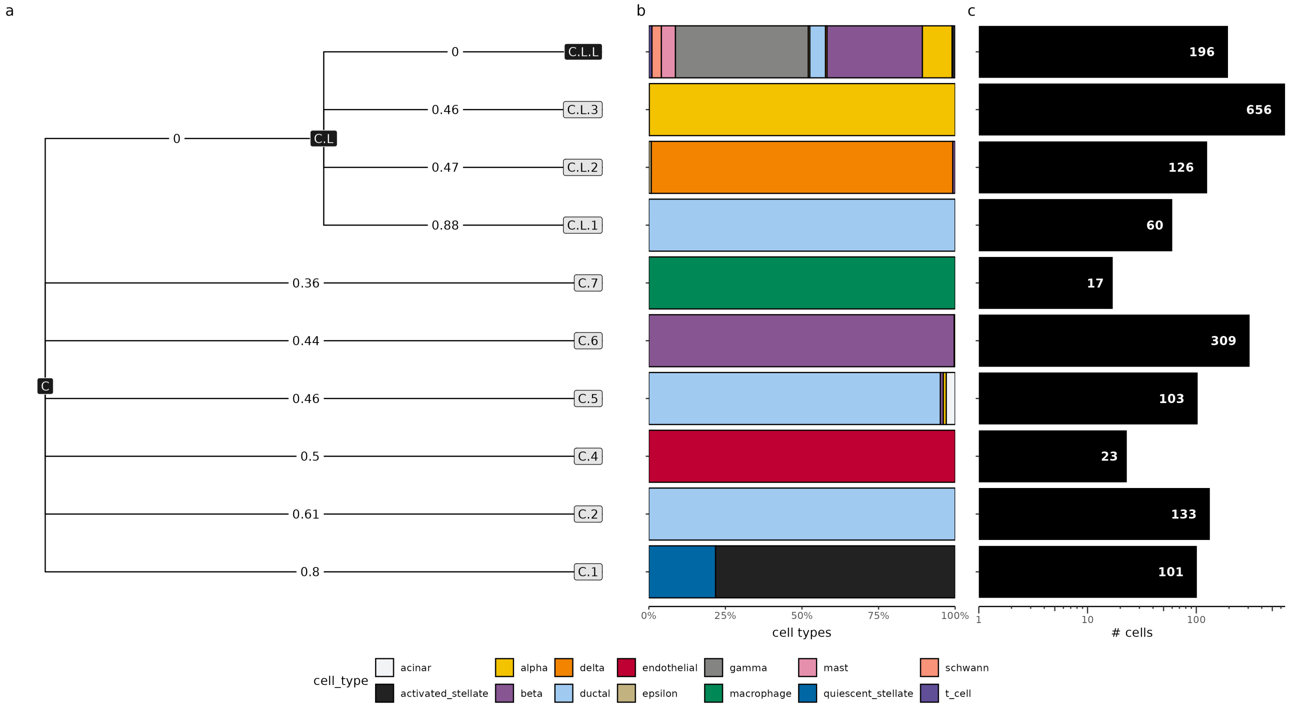

Supplementary Fig. 10. Clustering results of scEVE on the Baron_HumPan_2 dataset.

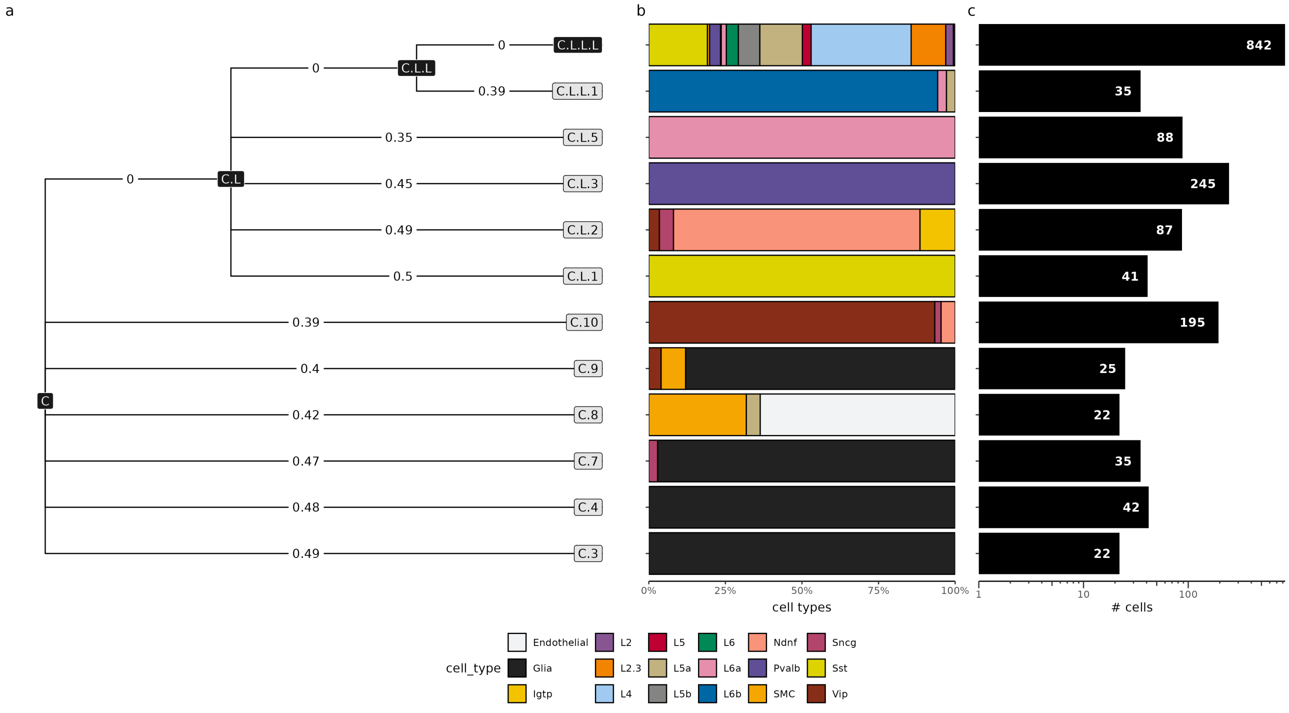

Supplementary Fig. 11. Clustering results of scEVE on the Tasic_MouBra dataset.

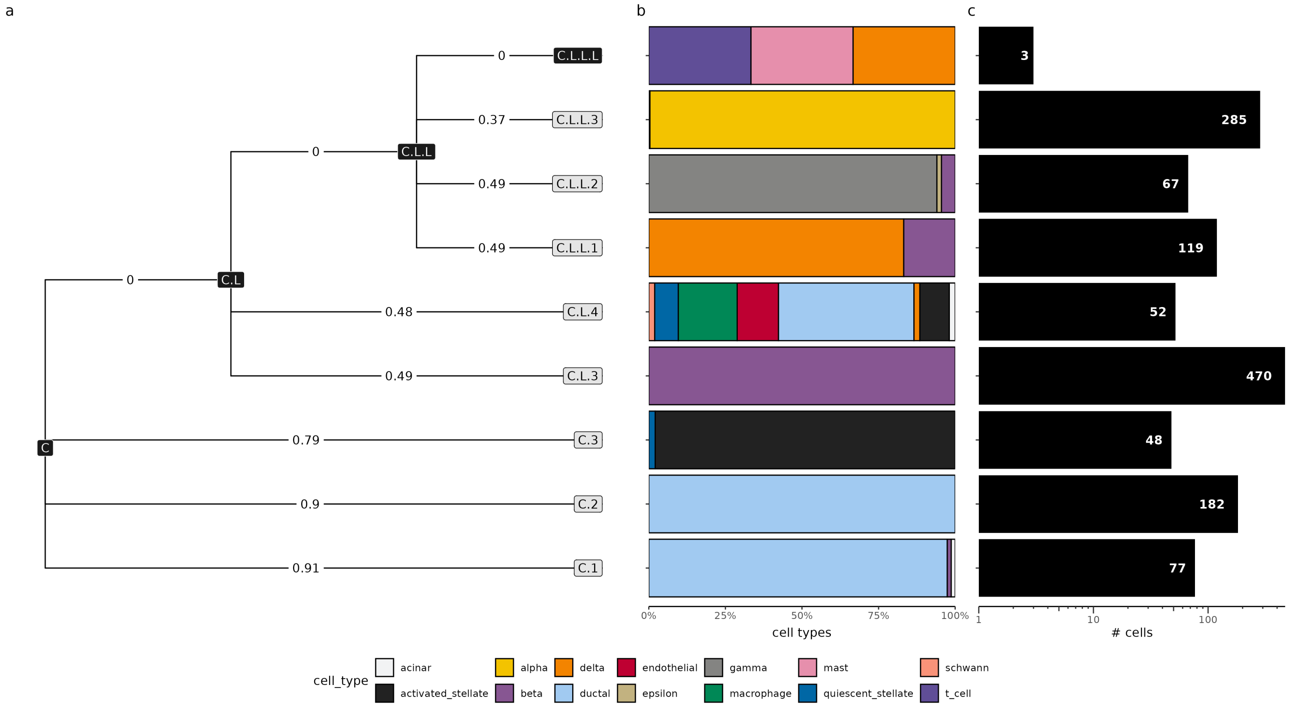

Supplementary Fig. 12. Clustering results of scEVE on the Baron_HumPan_4 dataset.

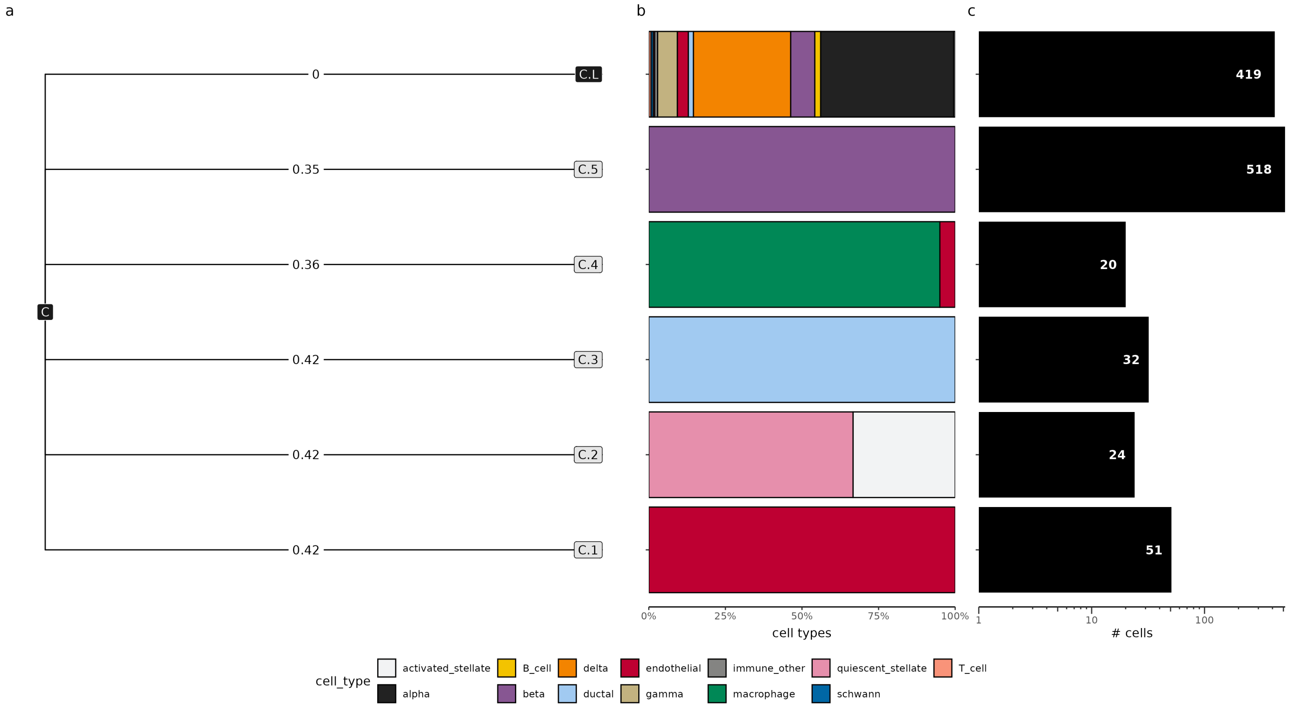

Supplementary Fig. 13. Clustering results of scEVE on the Baron_MouPan_2 dataset.

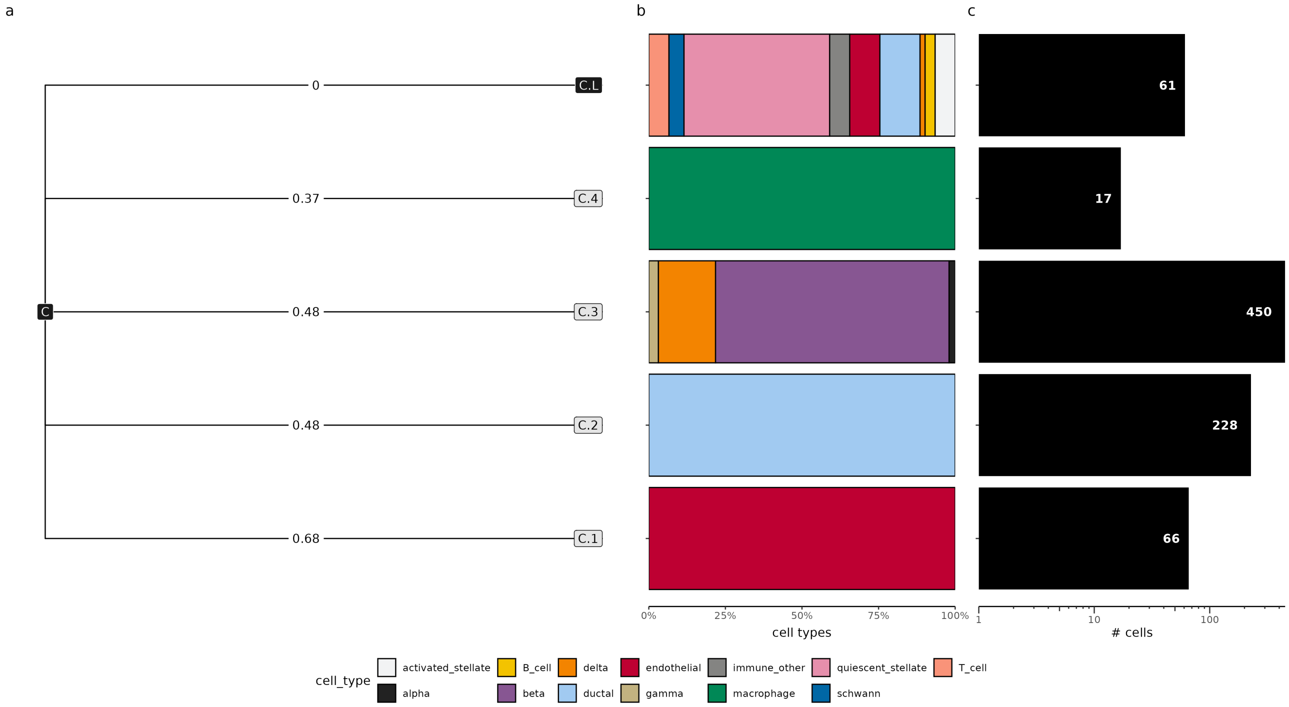

Supplementary Fig. 14. Clustering results of scEVE on the Baron_MouPan_1 dataset.

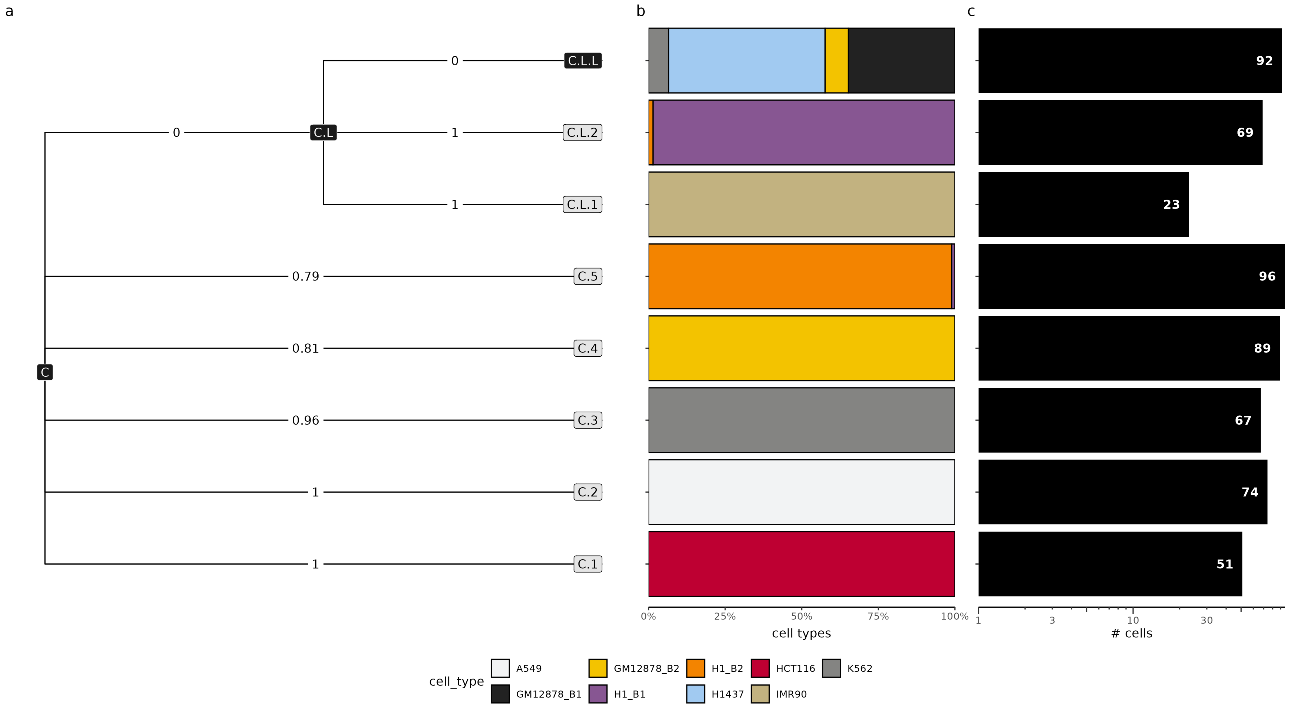

Supplementary Fig. 15. Clustering results of scEVE on the Li_HumCRC_a dataset.
